# A rapid live-cell ELISA for characterizing antibodies against cell surface antigens of *Chlamydomonas reinhardtii* and its use in isolating algae from natural environments with related cell wall components

**DOI:** 10.1186/s12870-014-0244-0

**Published:** 2014-09-25

**Authors:** Wenzhi Jiang, Sarah Cossey, Julian N Rosenberg, George A Oyler, Bradley JSC Olson, Donald P Weeks

**Affiliations:** Department of Biochemistry, University of Nebraska–Lincoln, 1901 Vine Street, Lincoln, NE 68588 USA; Division of Molecular, Cellular and Developmental Biology, Kansas State University, Manhattan, KS 66506 USA; Department of Chemical & Biomolecular Engineering, Johns Hopkins University, 3400 North Charles Street, Baltimore, MD 21218 USA; Synaptic Research, LLC, 1448 South Rolling Road, Baltimore, MD 21227 USA

**Keywords:** Live-cell ELISA, Camelid antibodies, Algae, Cell walls, V_H_H, Chlamydomonas, Chlorophyceae, Cell wall conservation, Nanobodies

## Abstract

**Background:**

Cell walls are essential for most bacteria, archaea, fungi, algae and land plants to provide shape, structural integrity and protection from numerous biotic and abiotic environmental factors. In the case of eukaryotic algae, relatively little is known of the composition, structure or mechanisms of assembly of cell walls in individual species or between species and how these differences enable algae to inhabit a great diversity of environments. In this paper we describe the use of camelid antibody fragments (VHHs) and a streamlined ELISA assay as powerful new tools for obtaining mono-specific reagents for detecting individual algal cell wall components and for isolating algae that share a particular cell surface component.

**Results:**

To develop new microalgal bioprospecting tools to aid in the search of environmental samples for algae that share similar cell wall and cell surface components, we have produced single-chain camelid antibodies raised against cell surface components of the single-cell alga, *Chlamydomonas reinhardtii*. We have cloned the variable-region domains (V_H_Hs) from the camelid heavy-chain-only antibodies and overproduced tagged versions of these monoclonal-like antibodies in *E. coli*. Using these V_H_Hs, we have developed an accurate, facile, low cost ELISA that uses live cells as a source of antigens in their native conformation and that requires less than 90 minutes to perform. This ELISA technique was demonstrated to be as accurate as standard ELISAs that employ proteins from cell lysates and that generally require >24 hours to complete. Among the cloned V_H_Hs, V_H_H B11, exhibited the highest affinity (EC_50_ < 1 nM) for the *C. reinhardtii* cell surface. The live-cell ELISA procedure was employed to detect algae sharing cell surface components with *C. reinhardtii* in water samples from natural environments. In addition, mCherry-tagged V_H_H B11 was used along with fluorescence activated cell sorting (FACS) to select individual axenic isolates of presumed wild relatives of *C. reinhardtii* and other Chlorphyceae from the same environmental samples.

**Conclusions:**

Camelid antibody V_H_H domains provide a highly specific tool for detection of individual cell wall components of algae and for allowing the selection of algae that share a particular cell surface molecule from diverse ecosystems.

**Electronic supplementary material:**

The online version of this article (doi:10.1186/s12870-014-0244-0) contains supplementary material, which is available to authorized users.

## Background

The cell walls of land plants and algae provide physical support and protection against various environmental factors and stresses. While much is known about plant cell walls [1], our knowledge of algal cell walls is more rudimentary [2,3]. Although it is known, for example, that cell walls of algae and land plants can contain abundant hydroxyproline-rich glycoproteins e.g., [4,5], studies of the composition and structure of algal cells walls and the diversity of cell wall components within and between algal species lag far behind that of land plants. Thus, detailed comparisons of cell wall compositions, synthesis and deposition between land plants and algae (and between different species of algae) are not presently possible. To help address this deficiency, we sought to develop techniques that would allow identification of cell surface-specific molecules not only in one particular alga, but also in closely related algal species in a variety of environmental locations. Monoclonal antibodies raised against such cell wall proteins, glycoproteins and other components have been used in the recent past as a powerful tool for allowing detection and characterization of plant and algal cell wall components [6,7] and have potential as a highly valuable tool for isolation of algae with shared cell surface constituents. An alternative approach that provides the same single-molecule specificity as conventional monoclonal antibodies involves use of camelid antibodies [8] that are composed of a single heavy chain molecule and used widely as highly specific, high affinity antibodies for numerous applications [9-12]. Genes encoding the single-domain antigen-binding fragment (V_H_H) of camelid heavy-chain-only antibodies [that we will refer to generically as V_H_Hs or, alternatively, single-domain antibodies (sdAbs) or nanobodies] can be cloned into bacteriophage-based expression vectors that allow a phage-display library of clones to be “panned” for V_H_Hs against a particular target antigen [13,14]. (Multiple targets can screened simultaneously in the initial panning). Individual cloned genes are modified to produce tagged V_H_H that can be readily detected during ELISA assays to measure their affinity for the target antigen or, for example, in the selection of algal species expressing the target antigen on their cell surface. As an initial proof-of-concept for this approach we chose to utilize *Chlamydomonas reinhardtii* (hereafter referred to as Chlamydomonas) as the alga whose cell wall is the most studied to date [3,5].

To generate camelid antibodies against Chlamydomonas antigens, we immunized alpacas with whole cell extracts of Chlamydomonas and prepared phage-display libraries of genes encoding variable-domain (V_H_H) regions of individual single-domain antibodies each having specific affinity to a particular epitope on an individual algal cell antigen [15]. From the phage-display library containing V_H_Hs raised against Chlamydomonas proteins and other immunogenic molecules, a number of phage clones were selected that bound well to the outer surface of live Chlamydomonas cells. Subsequently the V_H_H gene form each selected phage clone was subcloned into an *E. coli* overexpression vector. The V_H_H encoding sequence was cloned upstream and in frame with the coding region for an E-Tag peptide to allow facile detection of the E-tagged/V_H_H chimeric protein. Characterization of the individual E-tagged nanobodies overproduced in *E. coli* using standard enzyme-linked immunosorbent assays (ELISAs) showed that several of these clones bound with moderate to high affinity to proteins and other molecules from cell lysates of Chlamydomonas when these antigens were bound to the walls of wells in polystyrene microtiter plates [15].

Because each standard ELISA assay requires several hours to perform [14,16,17], we sought an equally accurate, but faster, more facile and economic means of determining the affinity with which V_H_Hs bound to Chlamydomonas cell surface molecules. Given that the initial selection of antibodies with specificity for the Chlamydomonas cell surface had been conducted with live Chlamydomonas cells, we reasoned that it might be possible to develop a modified ELISA procedure in which live cells provided the antigens needed for the assay. Instead of E-tagged sdAbs binding to proteins and other molecules immobilized on polystyrene surfaces to select high affinity V_H_Hs, we hypothesized that we could use a set number of Chlamydomonas cells (providing an excess of cell surface antigens) in individual microfuge tubes containing E-tagged V_H_H antibodies and then remove non-adhering nanobodies by multiple washing steps involving brief centrifugations and cell suspensions.

In their standard form [14,16-18], ELISAs have proven to be dependable and accurate methods for measuring antibody affinities for specific antigens and for providing estimates of antigen concentrations in samples associated with medical research and practice, agriculture, forensics and industry. An important limitation of the standard ELISA protocol is the time required for binding a target antigen to a solid matrix (generally the wall of wells in a polystyrene microtiter plate) and the multiple washing steps needed to remove unbound antibodies from the wells of the microtiter dish. In the present study, the standard ELISA protocol was recapitulated using a set of microfuge tubes each containing a set number of Chlamydomonas cells and that were inoculated with progressively increasing amounts of E-tagged V_H_Hs. The goal was to mimic corresponding antigen-saturated wells in microtiter plates used for standard ELISA assays. Subsequent steps involving incubation with secondary antibodies conjugated with horseradish peroxidase (HRP), addition of a non-chromogenic substrate and spectrophotometric analysis of the chromogenic product of the HRP reaction would be essentially identical to corresponding steps in the standard ELISA procedure.

A search of past literature revealed two early examples of development of live-cell ELISA assays for use with animal cells. The first [19] involved the use of various types of live human cancer and non-cancerous cells to screen for and characterize monoclonal antibodies with specificity for antigens present on the cancer cells but absent from the surface of non-cancerous cells of the same tissue type. The second [20] also utilized a live-cell ELISA to detect antigens specific to different types of cancer cells - in this case, bovine lymphosarcoma cells. More recent examples of live-cell ELISA using mammalian cells have been reviewed by Lourenço and Roque-Barreira [21]. Numerous examples exist of using cells killed by various fixation processes in whole-cell ELISA assays, but, as widely recognized, these methods suffer from the fact that the fixation processes involved often alter the structure and, therefore, the antigenicity of the surface molecules that are the targets of investigation [21]. Our goal in developing a live-cell ELISA analysis of algal cells was to offer the algal and microbiology communities a robust and facile new tool for detecting and roughly quantifying populations of micoorganisms bearing cell surface antigens of targeted interest.

Here we report success in developing a rapid, small-scale, live-cell ELISA assay for algae, demonstrate its equivalence to the standard ELISA procedure, employ it to measure the affinity of various V_H_Hs to components of the Chlamydomonas cell surface, and show that the high-affinity V_H_H B11 antibody binds specifically to Chlamydomonas and to other closely related Chlorophycean algae. We also provide visualization of the specificity of binding of V_H_H B11 to the Chlamydomonas cell surface by creating and employing V_H_H B11 green or red fluorescent proteins that brightly decorate the exterior of live Chlamydomonas cells, but not the surfaces of unrelated algae, during fluorescence microscopy. Finally, we employ the live-cell ELISA techniques and fluorescently-tagged V_H_H B11 antibodies to demonstrate the presence of wild Chlorophycean relatives of Chlamydomonas in environmental water samples and the isolation by fluorescence activated cell sorting of individual wild relatives of *C. reinhardtii* in those water samples.

## Results and discussion

### Analyses of candidate V_H_H nanobodies with the chlamydomonas live-cell ELISA

**Figure 1:**
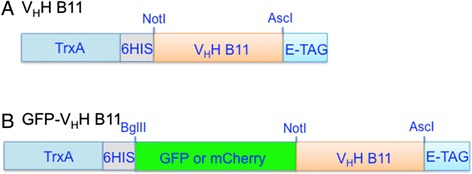
**Cassettes for over-expression in** ***E. coli*** **of the V**_**H**_ **H B11 gene encoding an antibody that recognizes a specific** ***C. reinhardtii*** **cell surface antigen. A**. V_H_H B11 cassette for expression of the V_H_H B11 fusion protein containing the Trx A protein at the N-terminus, an internal 6 × His tag, and an E-tag epitope at the C-terminus. **B**. GFP-V_H_H B11 cassette: identical to V_H_H B11 cassette except for insertion of a GFP or mCherry coding region immediately upstream and in-frame with the V_H_H coding region.

**Figure 2:**
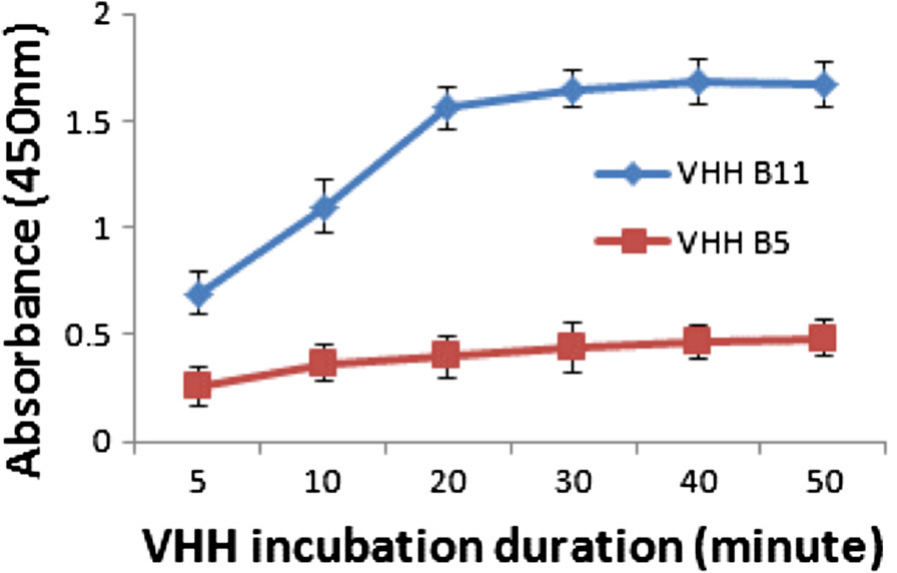
**Effect of incubation duration on the binding of V**_**H**_ **H B11 to living Chlamydomonas cells.** Colorimetric analysis of the effects of duration of incubation on the progression of binding of V_H_H B11 (blue line) at a concentration of 20 nM to living *C. reinhardtii* cells. BoNT V_H_H B5 (red line) binding to Chlamydomonas cells served as a negative control. Error bars represent standard deviation.

Overproduction of each candidate Chlamydomonas cell surface specific sdAb antibody was achieved by cloning the V_H_H coding region into the pET32b overexpression vector downstream of coding sequences for thioredoxin A and 6 × His (for recombinant protein purification) and upstream of the coding region of an E-tag epitope (Figure 1A). The latter allowed for recognition of the V_H_H by an E-tag-specific antibody conjugated to horse radish peroxidase (HRP) whose relative enzyme activity served as a measure of the quantity of sdAbs bound to a target antigen in a given assay. Each TrxA/6 × His/V_H_H/E-tag chimeric protein was tested for its affinity to antigens present (in excess) on the surface of Chlamydomonas cells in the rapid, small-scale, live-cell ELISA procedure described in detail in Methods. The key to the speed of this assay is that it requires less than 30 minutes for the binding of the added antibody to come to equilibrium (Figure 2) and each of two wash steps to remove unbound antibody is accomplished by a quick succession of microfuge centrifugation/cell resuspension steps that, together, consume only 4 minutes. Subsequent incubation with E-tag-specific and HRP conjugated secondary antibody, removal of unbound secondary antibodies by two centrifugation/cell resuspension steps, incubation with non-chromogenic 3,3′,5,5′-tetramethylbenzidine (TMB) and measurement of absorbance of the yellow reaction product at 450 nm all require 30–40 minutes. Based on experience from multiple experiments, this results in a total assay time of less than 1.5 hours. Standard ELISAs utilize overnight adsorption of antigens to the polystyrene wall of microtiter plate walls with additional manipulations consuming approximately 5 to 8 hours.

### Analyses of affinities of V_H_Hs to Chlamydomonas cell surface molecules

**Figure 3:**
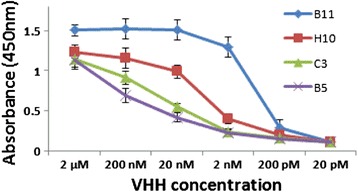
**Affinity of cell surface-specific V**_**H**_ **Hs to living** ***C. reinhardtii*** **cells.** Live-cell ELISA analyses comparing binding affinities to *C. reinhardtii* cells of various E-tag V_H_Hs (B11, blue line; H10, red line; C3, green line); and V_H_H B5 (a V_H_H binding specifically to a *Clostridium botulinum* BoNT/B holotoxin; negative control) purple line. Cells were incubated with serial dilutions of E-tag V_H_Hs at concentrations from 2 μM to 20 pM. E-tag V_H_H nanobodies attached to Chlamydomonas cells were detected using a HRP conjugated E-tag antibody that reacted with TMB (3,3′,5,5′-tetramethylbenzidine) to measure amounts of V_H_H bound to cell surface antigens. Error bars represent standard deviation.

Three cell surface-specific sdAbs, V_H_H B11, V_H_H H10 and V_H_H C3 were analyzed with the Chlamydomonas live-cell ELISA protocol. Two nanobodies (V_H_H B11 and V_H_H H10) displayed EC_50_ levels of 10 nM or less, with V_H_H B11 exhibiting the highest affinity EC_50_ < 1 nM (Figure 3). V_H_H C3, displayed markedly higher EC_50_ values and only slightly lower than that obtained with a sdAb raised against *Clostridium botulinum* BoNT/B holotoxin – the V_H_H used throughout these studies as a negative control (Figure 3). Importantly, results of experiments using the Chlamydomonas live-cell ELISA produced nearly identical EC_50_ values for V_H_H B11, V_H_H H10, and V_H_H C3 and V_H_H BoNT/B (i.e., 0.5 nM, 10 nM, 50 nM and 1000 nM, respectively) as obtained with a standard ELISA in analyses employed during our original studies [15].

### Specificity of V_H_H B11 for chlorophyceaen algae

**Figure 4:**
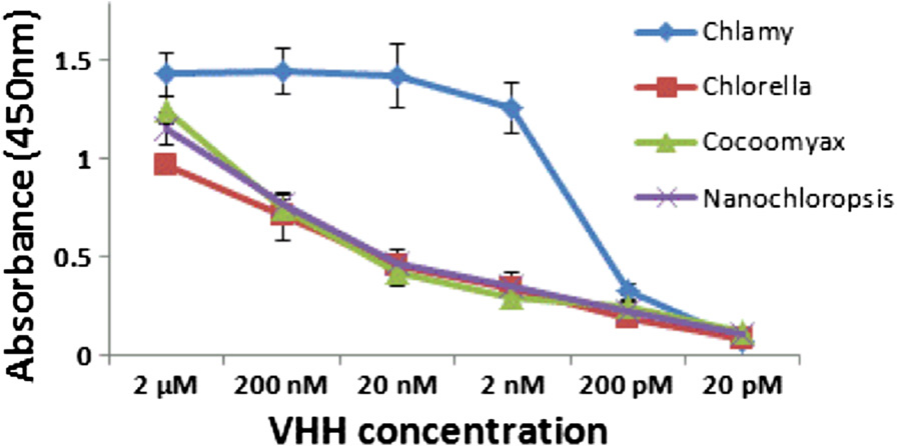
**Affinity of V**_**H**_ **H B11 to Chlamydomonas and other algal cells.** Live-cell ELISA analyses comparing the binding affinity of V_H_H B11 to living *Chlamydomonas reinhardtii* (cc124) cells (blue line) and other living algae cells (Chlorella, red line; Nannochloropsis, purple line; Coccomyxa, green line). Error bars represent standard deviation.

To determine if V_H_H B11 recognizes all algae, or is restricted to Chlorophycean algae, we performed live-cell ELISA assays on two Heterokonts (aka Stramenopiles), *Nannochloropsis oceanica* and *Thalassiosira pseudonana*. When substituted for Chlamydomonas in the live-cell ELISA, none of these algae exhibited affinities above background levels (i.e., affinities exhibited by V_H_H BoNT/B) (Figure 4). Likewise, V_H_H H10 showed affinity only for Chlamydomonas when assayed in an analogous experiment (data not shown). Interestingly, we repeated the live-cell ELISAs with the Chlorophycean alga *Coccomyxa subellipsoidea* and did not observe significant affinity. The genome size of *C. subellipsoidea* that resides in cold polar regions is greatly reduced in size compared to its close Chlorophycean relatives found in temperate climates [22]. One of the key families of Chlorophycean genes lost in its genome are those encoding glycosyl phosphatidyl inositol transamidase that attach cell surface proteins to the plasma membrane [22]. Whether it is the loss of this gene or another gene that may be responsible for the lack of V_H_H B11 interaction with the cell wall of this Chlorophycean species will need to await future determination of the identity of the antigen to which V_H_H B11 binds. However, the ability of the V_H_H B11 antibody to detect differences between cell walls of closely related Chlorophyceans from different environments points to the usefulness of camelid antibodies and monoclonal antibodies in helping to define specific differences in cell wall composition between different algae and determining how these differences contribute to ecological adaptation.

In regard to specificity of V_H_H B11 for Chlorophyceaen algae, it should be noted that in studies described below in which several samples of water from natural environments were tested, a number of the samples containing large numbers and varieties of algae tested negative using either V_H_H B11 or V_H_H H10 – again suggesting strong selectivity of these two sdAbs for the cell surface of Chlamydomonas or Chlamydomonas-related algae and not to distantly related algae.

### Saturation of V_H_H B11 binding with increasing Chlamydomonas cell densities

**Figure 5:**
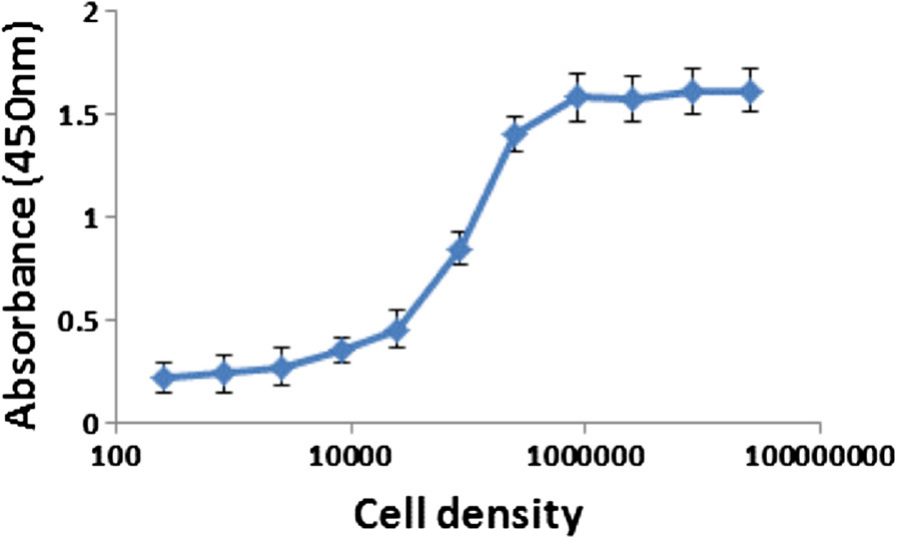
**Effect of cell density on the binding of V**_**H**_ **H B11 to living Chlamydomonas cells.** Colorimetric analysis the effects of cell density on the binding of V_H_H B11 (blue line) to living *C. reinhardtii* cells. Cells at different densities were incubated with V_H_H B11 at a concentration of 20 nM. Error bars represent standard deviation.

During initial experiments to ensure that an excess of cell surface antigens were present in our live-cell ELISAs, a set concentration of V_H_H B11 (20 nM) was used in each of a set of microfuge tubes into which progressively increasing concentrations of live Chlamydomonas cells were added (i.e., from 2.5 × 10^2^ cell/0.5 mL to 2.5 × 10^7^ cells/0.5 mL). The results of this experiment indicated that slightly less than 10^6^ cells/0.5 mL were needed to cause all V_H_H B11 molecules to be associated with cell surface antigens (Figure 5). Thus, for subsequent live-cell assays, Chlamydomonas cell concentrations of approximately 10^6^ cells/0.5 mL were employed.

### Modification of the live-cell ELISA for detection of algae in environmental samples

**Figure 6:**
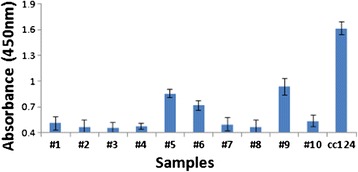
**ELISA test for binding of V**_**H**_ **H B11 to Chlamydomonas and to other algal cells in pond water samples.** Colorimetric analyses comparing the binding affinity of V_H_H B11 to living *C. reinhardtii* (cc124) cells and to mixtures of other living algae in 10 independent pond water samples. Error bars represent standard deviation.

Using a modification of our new live-cell ELISA protocol we also developed a rapid, small-scale method for obtaining rough estimates of populations of Chlamydomonas-related cells (i.e., those displaying the surface antigen to which V_H_H B1 binds) in samplings of algae from natural settings. In these assays, the algal samples were concentrated by centrifugation and resuspended in a mixture of V_H_H B11 and reagents to a cell density the same as used in the Chlamydomonas live-cell ELISA procedure. After two washings, cells were subjected to the prescribed protocols (see descriptions above and Methods) for incubation with secondary HRP conjugated E-tag antisera and measurements of enzyme activity. Evaluation using the live-cell ELISA analysis of ten independent environmental water samples allowed rapid identification of three of these samples as containing appreciable numbers of algae capable of binding V_H_H B11 (Figure 6).

### GFP/mCherry V_H_H B11 chimeras and their use in identifying novel *C. reinhardtii*-related unicellular chlorophycean aglae

**Figure 7:**
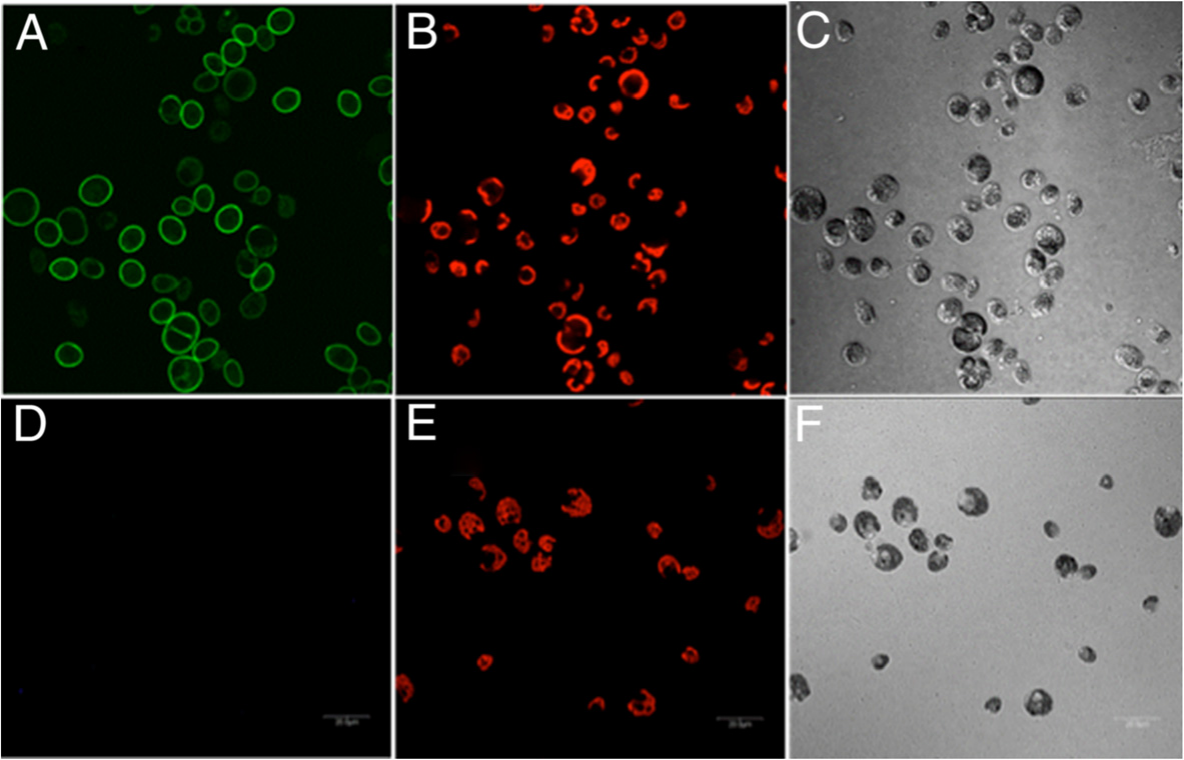
**Confocal microscope images of wild type** ***C. reinhardtii*** **(cc124) incubated with the GFP-V**_**H**_ **H B11 chimeric nanobody. A)** Cells detected in the GFP fluorescence channel displaying specific staining of the cell walls. **D)** Cells incubated with a GFP-V_H_H B5 (negative control) showing no fluorescence. **A** and **D**: GFP fluorescence channel, **B** and **E**: chloroplast auto-fluorescence channel; **C** and **F**: phase contrast images of cells.

Further analyses of algae in environmental samples took advantage of our earlier described [15] coupling of the coding region of the green fluorescent protein (GFP) to the 5’ terminus of the V_H_H B11 coding region (Figure 1B) to produce a GFP/V_H_H B11 chimera. This chimera could then be used to demonstrate specific binding of the antibody to the cell surface of Chlamydomonas using confocal microscopy (Figure 7A). Incubation of Chlamydomonas with GFP V_H_H B5 anti-botulinum toxin nanobody (negative control) produced no fluorescently stained cells (Figure 7D). Incubation of *Nannochloropsis oceanica, Coccomyxa subellipsoidea,* and *Thalassiosira pseudonana* with the GFP/V_H_H B11 produced no GFP signal (data not shown).

**Figure 8:**
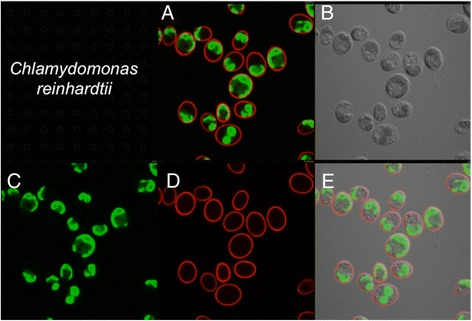
**Confocal microscope images of** ***C. reinhardtii*** **incubated with mCherry V**_**H**_ **H B11 chimeric antibody. A)** Merged image from **C** (chlorophyll fluorescence; pseudo green) and **D** (mCherry red fluorescence). **B)** Phase contrast image of cells. **E)** Merged images from **B**, **C** and **D**.

**Figure 9:**
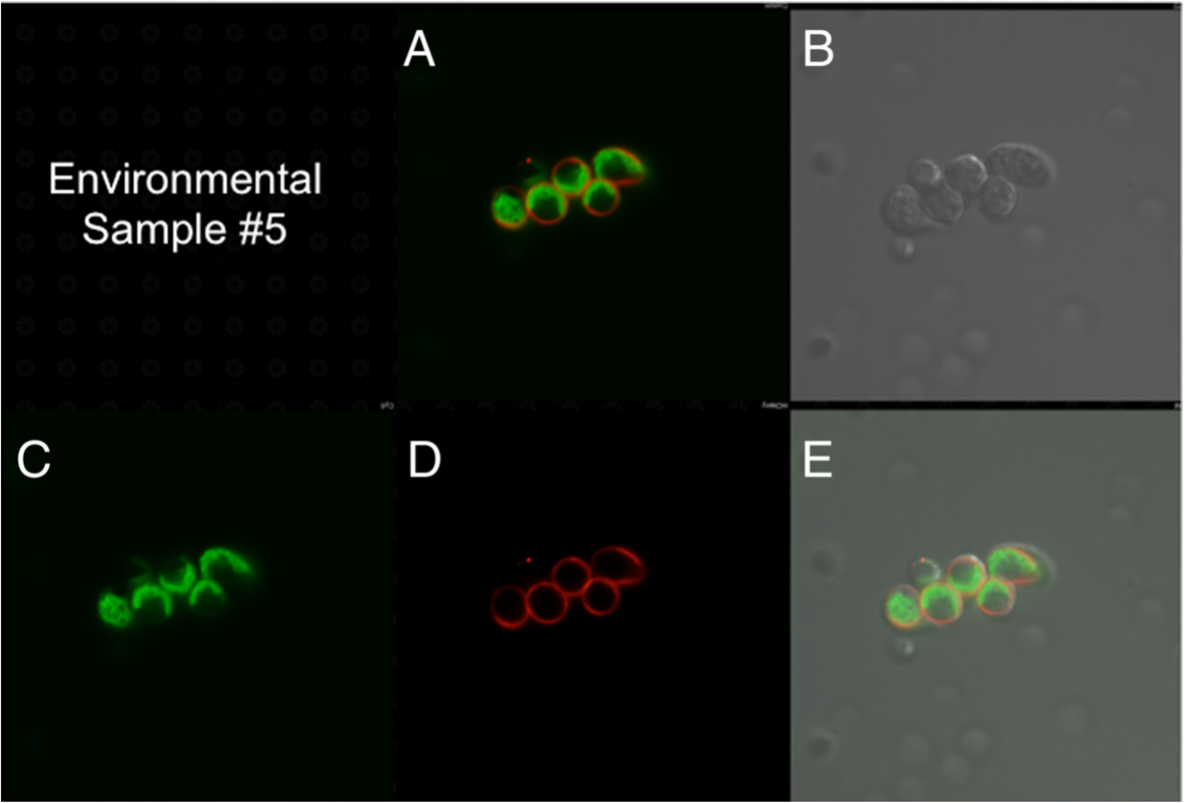
**Confocal microscope images of sample #5 cells incubated with mCherry V**_**H**_ **H B11 chimeric nanobody. A)** Merged image from **C** (chlorophyll fluorescence; pseudo green) and **D** (mCherry red fluorescence). **B)** Phase contrast image of cells. **E)** Merged images from **B**, **C** and **D**.

**Figure 10:**
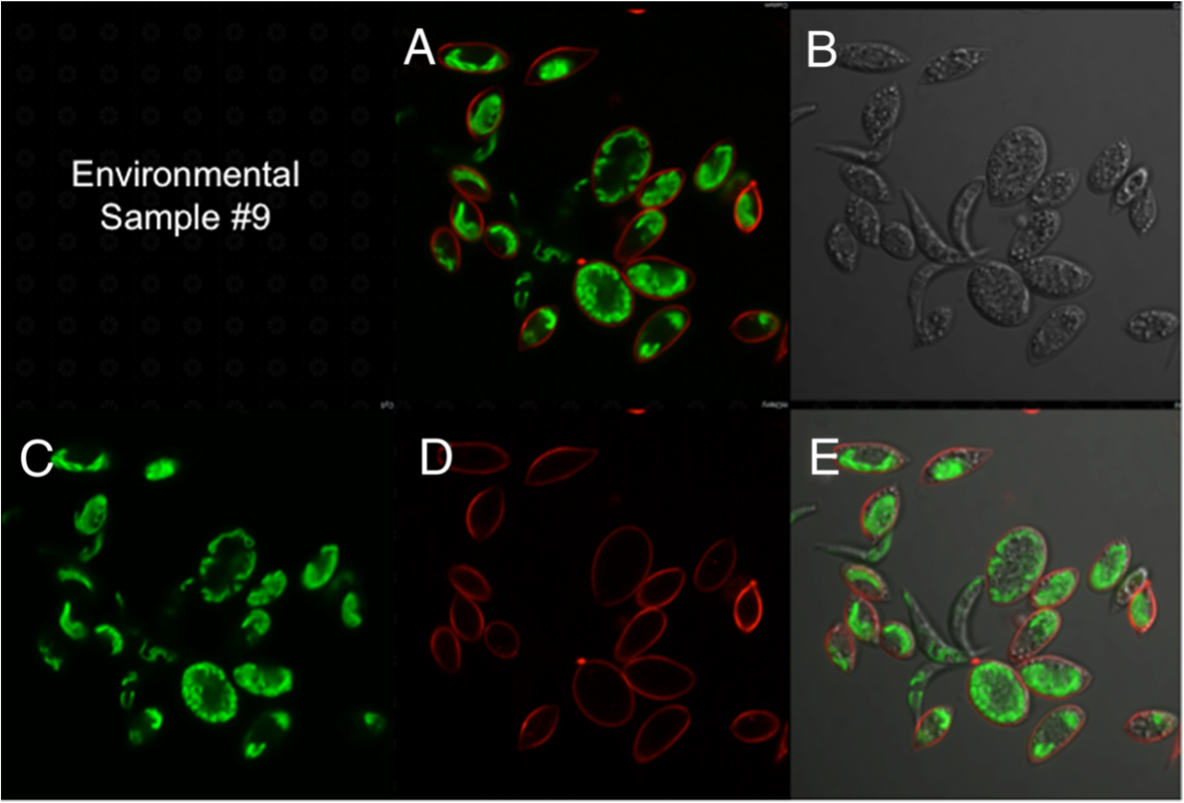
**Confocal microscope images of sample #9 cells incubated with mCherry V**_**H**_ **H B11 chimeric nanobody. A)** Merged image from **C** (chlorophyll fluorescence; pseudo green) and **D** (mCherry red fluorescence). **B)** Phase contrast image of cells. **E)** Merged images from **B**, **C** and **D**.

**Figure 11:**
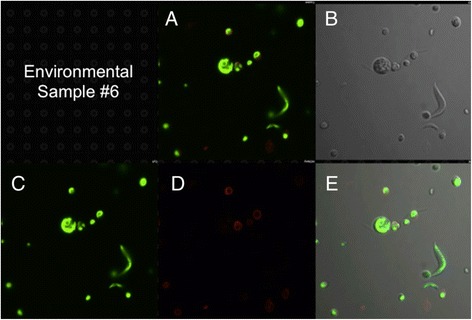
**Confocal microscope images of sample #6 cells incubated with mCherry V**_**H**_ **H B11 chimeric nanobody. A)** Merged image from **C** (chlorophyll fluorescence; pseudo green) and **D** (mCherry red fluorescence). **B)** Phase contrast image of cells. **E)** Merged images from **B**, **C** and **D**.

To search for *C. reinhardtii* or closely related Chlorophyceae species in the water samples discussed above, we mixed algae in the samples with an mCherry/V_H_H B11 chimera prior to examination by confocal microscopy. While seven samples failed to yield cells capable of binding the mCherry/V_H_H B11 nanobody, three water samples displaying the highest ELISA values (Figure 6: #5, #6 and #9) contained a subpopulation of algal cells capable of binding with mCherry/V_H_H B11. When compared with binding of mCherry/V_H_H B11 to *C. reinhardtii* cell walls (Figure 8), two algae bound to a nearly equal extent (Figures 9 and 10), while the third bound to a distinctly lower extent (Figure 11).

**Figure 12:**
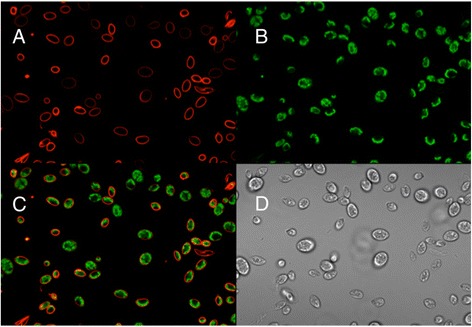
**Confocal microscope images of a presumed wild relative of Chlamydomonas (#9-2i) isolated from environmental sample #9 by flow cytometry after staining with mCherry/VHH B11 nanobody.** Single cells separated by flow cytometry were cultured on solid TAP medium prior to resuspention in liquid medium and confocal microscopic analysis. **A)** mCherry staining of cell walls. **B)** Chlorophyll fluorescence (pseudo green color). **C)** Merged images from **B** and **C**. **D)** Phase contrast image of cells.

As further demonstration of the utility of the mCherry/V_H_H B11 nanobodies, we subjected cells from environmental water sample #9 to fluorescence activated cell sorting after incubation with mCherry/V_H_H B1. In so doing, we were able to capture single cells (e.g., cell isolate #9-2i; Figure 12) to which the mCherry-labeled nanobody was bound and culture them on solid medium in preparation for taxonomic classification based on DNA sequencing of their 18S ribosomal RNA genes (described below).

### Species identification of chlorophycean relatives that react with the V_H_H B11 sdAb

Having identified three strains that strongly react with V_H_H B11 in environmental water samples, we identified the algae by sequencing their ribosomal internal transcribed spacer regions (ITS1 and ITS2) [23]. First, to provide additional insurance that each of the three environmental isolates were axenic, we performed multiple rounds of antibiotic washing, dilution, and plating for single clones on tris-phosphate (TP) plates. Three or more decontaminated clones of each isolate were pooled prior to ITS analysis.

**Figure 13:**
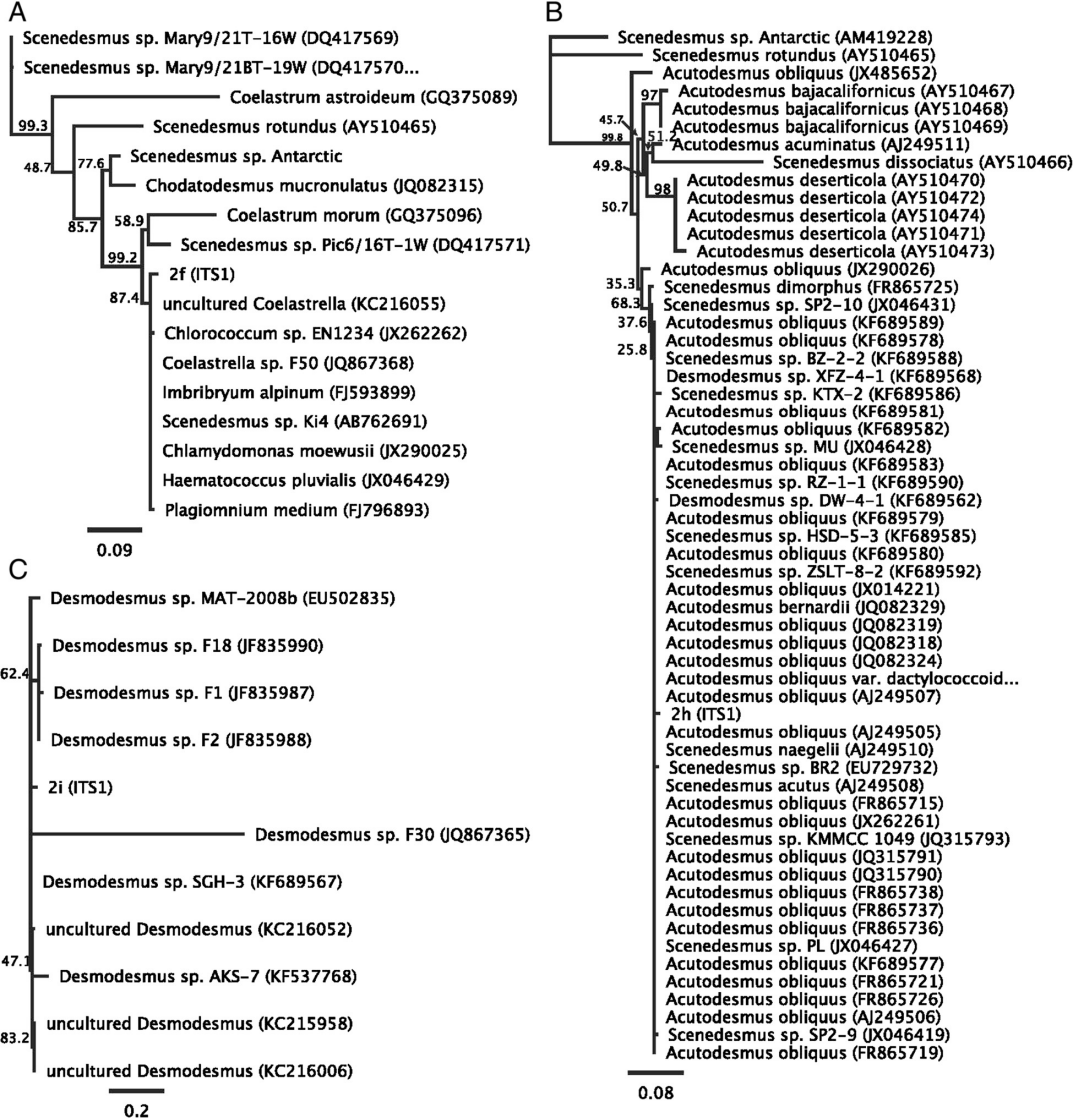
**Phylogenetic tree of environmental isolates 2f, 2h and 2i.** Representative maximum likelihood phylogenies for the three environmental isolates 2f **(A)**, 2h **(B)** and 2i **(C)** based on ribosomal DNA ITS1 and ITS2 phylogenies. Shown are their closest subfamily members. Full phylogenetic analyses are shown in Additional file 1: Figures S5 and S6. Bootstrap values, when available are indicated at each node.

After amplification and sequencing of the ITS1 and ITS2 regions and phylogenetic analysis, isolate 2i phylogenetically clusters with several *Desmodesmus* species, where its ITS2 sequence demonstrate it is *D. pleiomorphus* (Figure 13). Interestingly, this is one of the few unicellular biflagellate species of *D. pleiomorphus* that has been described [24]. Likewise, strain 2 h phylogenetically clusters with *Scenedesmus obliquus* another taxonomically distinct group of unicellular bi-flagellate algae [25,26]. Interestingly, the *Scenedesmus* genus was originally morphologically characterized as being multicellular sheets of cells [27]. However with improved molecular phylogenetic techniques, many unicellular bi-flagellates previously placed in other groups have been transferred to *Scenedesmus* and its *Desmodesmus* sub-group [26].

Strain 2f is unique because it phylogenetically clusters with a group of environmental isolates found to be in close association with Bryophytes (Figure 9). Member of its clade include *Coelastrella* and *Scenedesmus* [26], as well as several mis-identified unicellular bi-flagellate algae (attributed as *C. moewussi,* though this group is far removed from the Chlamydomonacales (Figure 13 and Additional file 1: Figures S1 and S2). Because its closest relative has been positively identified as *Coelastrealla*, we currently classify this strain as such. Interestingly, taking a broad view of the phylogeny of these three novel environmental isolates demonstrates that V_H_H B11 broadly binds to cell-wall proteins found in unicellular Chlorophycean algae (Additional file 1: Figures S1 and S2). This demonstrates the broad usefulness of this antibody as a tool for identifying novel unicellular algae, but also suggests broad conservation of the cell wall amongst distantly related unicellular Chlorophycean algae.

DNA sequences of 18S ribosomal RNA gene ITS1 and ITS2 regions used in these studies for construction of phylogenetic maps have been deposited in GenBank and accession numbers are listed in Additional file 1: Table S1.

Future studies will focus on use of the V_H_H B11 nanobody to aid in the purification and molecular characterization of the target antigen from Chlamydomonas and the three different algal strains described here. The long-term goal will be to use a similar approach for isolation and characterization of additional cell wall/cell surface components that will allow not only comparisons of cell wall composition between related algae but also between cell walls of land plants and the algae from which they were evolutionarily derived.

A significant advantage of the live-cell ELISA procedure is that it allows interaction of V_H_Hs with cell surface antigens in their native state. This represents a significant improvement compared to standard ELISA procedures in which antigens are adsorbed to the polystyrene surface of microtiter plate wells, a step that often results in protein denaturation. A search of the literature has revealed no previous use of standard ELISAs or live-cell ELISAs to identify algae with shared cell wall components. Thus, the present study provides the research community with a facile new means for accomplishing this task. There are obvious limitations to the methods as presently described. For example, not all cell surface components will posses sufficient antigenicity to elicit a strong antibody response in immunized animals and, even if tight binding antibodies are obtained, there may be algae in which the target antigen is produced in very low quantities or may produce target antigens that are buried or masked within the cell wall.

## Conclusions

Together, the experimental results presented here demonstrate the ability of V_H_H B11 and mCherry-tagged V_H_H B11 to allow detection, isolation and identification of algal cells from various ecosystems that share cell wall and cell surface components with Chlamydomonas. These results point the way to future research aimed at discovery of additional cell wall/cell surface components shared by Chlorophycean algae and to the initiation of detailed biochemical, molecular and genetic studies of these molecules. More generally, use of the live-cell ELISA assay described here and the production of highly specific antibodies, such as the V_H_Hs employed in the present study, have the potential to greatly facilitate future searches of the natural environment for particular species of algae and other microorganisms of interest to a broad range of laboratories around the world.

## Methods

### Chemicals and biologicals

E-Tag Antibody (HRP conjugated) was purchased from Bethyl Laboratories Inc. (Catalog No. A190-132P). TMB (3,3′,5,5′-Tetramethylbenzidine (Liquid Substrate System for ELISA) was provided by Sigma (Catalog No. T0440). Protein concentrations were measured using a Bio-Rad Protein Assay (Catalog No. 500–0005).

### Environmental water sample preparation

Environmental water samples of 10 mL each were collected from the Holmes Lake area and other public and private ponds in Lancaster county, and Lincoln, NE. Collected cells were maintained in TP medium (TAP medium lacking acetate) in light under 3% CO_2_ with shaking at 100 RPM. ELISA analyses and fluorescence confocal microscopy were performed as described below. Single algal cells binding the GFP-V_H_H B11 were isolated using a BD FACS Aria flow cytometer.

### V_H_H expression vectors

Three surface binding V_H_H cDNA clones [15] in JSC phagemid vectors (GenBank Accession Number: EU109715) were cut with NotI/AscI and DNA fragments were migrated into a pET32b backbone pre-engineered to contain an E-Tag and NotI/AscI cloning sites. The resulting V_H_H protein products contained a N-terminal thioredoxin (Trx A) fusion partner, an internal 6 × His tag, and a C-terminal E-tag. Using these expression vectors as backbone, a GFP or mCherry coding region was fused directly to the N-terminus of the V_H_H coding region to allow production of fluorescent versions of V_H_Hs for confocal microscopy assays. To accomplish this, a GFP coding region (a synthetic construct encoding monomeric GFP fluorescent protein gene, Accession Number AAC53663) and a mCherry coding region (a synthetic construct encoding monomeric mCherry fluorescent protein gene, Accession Number AY678264) were inserted at BglII-NotI cutting sites in the V_H_H expression vectors, in such a way that the resulting V_H_H fluorescence protein products contained a N-terminal thioredoxin (Trx A) fusion partner, an internal 6 × His tag followed by a GFP or a mCherry fluorescence protein and a C-terminal E-tag.

### Expression and purification of V_H_H fusion proteins

*Escherichia coli* strain BL21(DE3) bearing the V_H_H fusion gene in pET32b was grown in LB media at 37°C with shaking until reaching an OD_600_ of 0.6. Expression of the recombinant protein was induced with 1 mM IPTG at 20°C for 20 hrs. The bacterial cells were harvested by centrifugation at 5000 × *g* for 15 min and resuspended in ice-cold lysis buffer [50 mM sodium phosphate (pH 8.0), 300 mM NaCl, 10 mM imidazole, 1 mM PMSF, and Protease Inhibitor Cocktail for use with bacterial cell extracts (Sigma, P8465)]. The re-suspended cells were treated with lysozyme at the concentration of 1 mg/mL for one-half hour before sonication at 4°C with a Sonics & Materials sonicator, Model VCX 600 (Sonics and Materials Inc, Danbury, CT, USA) at an amplitude of 30% in 9.9 s bursts with 9.9 s resting periods for 15 min. The sonicated cell lysate was clarified by centrifugation at 20,000 × *g*. The supernatant was loaded onto a Ni^2+^–NTA metal-affinity resin and washed with buffer containing 50 mM sodium phosphate (pH 8.0), 300 mM NaCl and 20 mM imidazole. Bound protein was released with elution buffer containing 50 mM sodium phosphate (pH 8.0), 120 mM NaCl and 250 mM imidazole. The eluted protein was dialyzed against 50 mM Tris (pH 7.5). The final protein concentration was determined using Bradford's reagent (Bio-Rad, Hercules, CA). Purity of the V_H_H fusion protein was determined by analysis on an overloaded, Coomassie-stained, SDS-PAGE. Only freshly prepared V_H_H fusion proteins were used for affinity assays.

### Live-cell V_H_H ELISA

For live-cell V_H_H ELISAs, 100 μL of a *C. reinhardtii* (CC124) culture or other algae cells at a density of approximately 10^7^ cells/mL was transferred into a 1.5 mL centrifuge tube, centrifuged at 6000 × g for 2 min, and resuspend in 500 μL TAP medium containing 1% dry milk (filter sterilized). Cells were shaken slowly under light for 5 min before addition of V_H_H at the desired final concentration. As controls, similar incubations with live Chlamydomonas cells were conducted in the presence of a V_H_H raised against *Clostridium botulinum* (BoNT V_H_H B5; 9). After incubation of the V_H_H protein with cells for 25 min under light, cells were collected by centrifugation, washed twice in 700 μL TAP medium and transferred to 500 μL TAP medium containing the equivalent of 0.025 μL of undiluted E-Tag antibody. Cells were incubated in the light with the E-Tag antibody for 25 min as described above. After centrifugation and two washes with TAP medium, cells were re-suspend in 100 μL TMB and mixed well. After 5 minutes, 100 μL of 1 N HCI was added to terminate the reaction. A buffer control was made by mixing 100 μL each 1 N HCI and TMB. Cells were pelleted by centrifugation at 13,000 × *g* for 1 min and the absorbance of the supernatant was measured at a wavelength of 450 nm.

### Fluorescence confocal microscopy

For analysis of binding of GFP- or mCherry-tagged nanobodies to the cell surface of *C. reinhardtii* and other algae using confocal microscopy, cells in 0.5 mL of cell culture at saturation density were collected by centrifugation at 5000 × *g* for 2 minutes in a 1.5 mL centrifuge tube. Cell pellets were re-suspended in 0.5 mL TAP medium containing 1% non-fat dry milk and then shaken for 15 min in light. Cells were washed twice with TAP medium and re-suspended in 0.5 mL TAP medium containing 1% non-fat dry milk, followed by the addition of chimeric mCherry or GFP V_H_H B11 nanobody to the desired final concentration, typically 30 nM. Fluorescence BoNT V_H_H B5 served as negative control. After shaking for 30 min in light, cells were washed twice with TAP medium. Cells were then examined by confocal fluorescence microscopy using a Nikon ECLIPSE 90i system at 1000× magnification. The excitation wavelength was set at 561.5 nm and the emission wavelength at 570-620 nm for mCherry fluorescence, 448 nm and 500–550 nm for GFP fluorescence and at 641 nm and 662-737 nm for chlorophyll auto-fluorescence to ensure no cross talk between different fluorescence channels.

### Taxonomic identification of environmental isolates

Environmental samples were initially maintained xenically, however, to taxonomically classify them, they were made axenic by ten alternating rounds of washing with sterile TP medium supplemented with 800 μg/mL carbenecillin, 5 μg/mL ciprofloxacin, 50 μg/mL chloramphenicol, 5 μg/mL trimethoprim and 0.1% tween-20, followed by centrifugation at 100 *g* for 2 minutes. After centrifugation, samples were top illuminated with 20 μE of light for 5 minutes, then the supernatant containing algae was removed and centrifuged at 1000 *g* for 5 minutes, the supernatant was discarded, while pelleted algal cells were collected. After washing and differential centrifugation, algal cells were serially diluted and plated on TP agar plates. Single colonies were picked into fresh media and tested for the presence of contaminating organisms by examination with microscopy and by replica plating on TAP agar supplemented with 5% yeast extract. Three independent clones were randomly chosen for taxonomic identification.

Genomic DNA was prepared from each independent clone with a plant specific spin column DNA preparation kit (Omega Biotek Plant EZNA). The ITS1 and ITS2 ribosomal spacer regions were independently amplified in two independent PCR reactions with Phusion DNA polymerase using primers for ITS1 [GGGATCCGTTTCCGTAGGTGAACCTGC (forward) and GCTGCGTTCTTCAGCGAT (reverse)] and for ITS2 [GGGATCCATATGCTTAAGTTCAGCGGGT (forward) and GCATCGATGAAGAACGCAGC (reverse)]. PCR products of the expected size were pooled and sub-cloned (Thermo pJECT), and three independent clones were sequenced. For all three strains, each of the independent algal and PCR product clones produced identical sequences. The ITS1 and ITS2 sequences were used to search the NCBI database by BLAST for closely related sequences. These sequences were aligned by MUSCLE [28]. Phylogenies were determined with a HKY85 substitution model using maximum likelihood in PhyML [29] with 100 rounds of bootstrap support.

## Additional file

Additional file 1:
**Supplementary Figures and Table.** **Figure S1.** Phylogenetic tree of environmental isolates 2f, 2h and 2i based on ITS 1 ribosomal DNA sequence comparisons. **Figure S2.** Phylogenetic tree of environmental isolates 2f, 2h and 2i based on ITS 2 ribosomal DNA sequence comparisons. **Table S1.** DNA sequences used for phylogenetic analyses.
