# Supplementary material for: A rapid live-cell ELISA for characterizing antibodies against cell surface antigens of Chlamydomonas reinhardtii and its use in isolating algae from natural environments with related cell wall components
Source: BMC Plant Biol. 2014 Sep 25;14:244. doi: 10.1186/s12870-014-0244-0 (PMC4181299; doi:10.1186/s12870-014-0244-0)
Supplement: Additional file 1: — Supplementary Figures and Table. Figure S1. Phylogenetic tree of environmental isolates 2f, 2h and 2i based on ITS 1 ribosomal DNA sequence comparisons. Figure S2. Phylogenetic tree of environmental isolates 2f, 2h and 2i based on ITS 2 ribosomal DNA sequence comparisons. Table S1. DNA sequences used for phylogenetic analyses. [file 12870_2014_244_MOESM1_ESM.docx]

**Additional Files - Supporting Data**

**Figure S1**. Phylogenetic tree of environmental isolates 2f, 2h and 2i based on ITS 1 ribosomal DNA sequence comparisons.

**Figure S2**. Phylogenetic tree of environmental isolates 2f, 2h and 2i based on ITS 2 ribosomal DNA sequence comparisons.

**Table S1**. GenBank accession numbers for DNA sequences used for phylogenetic analyses.


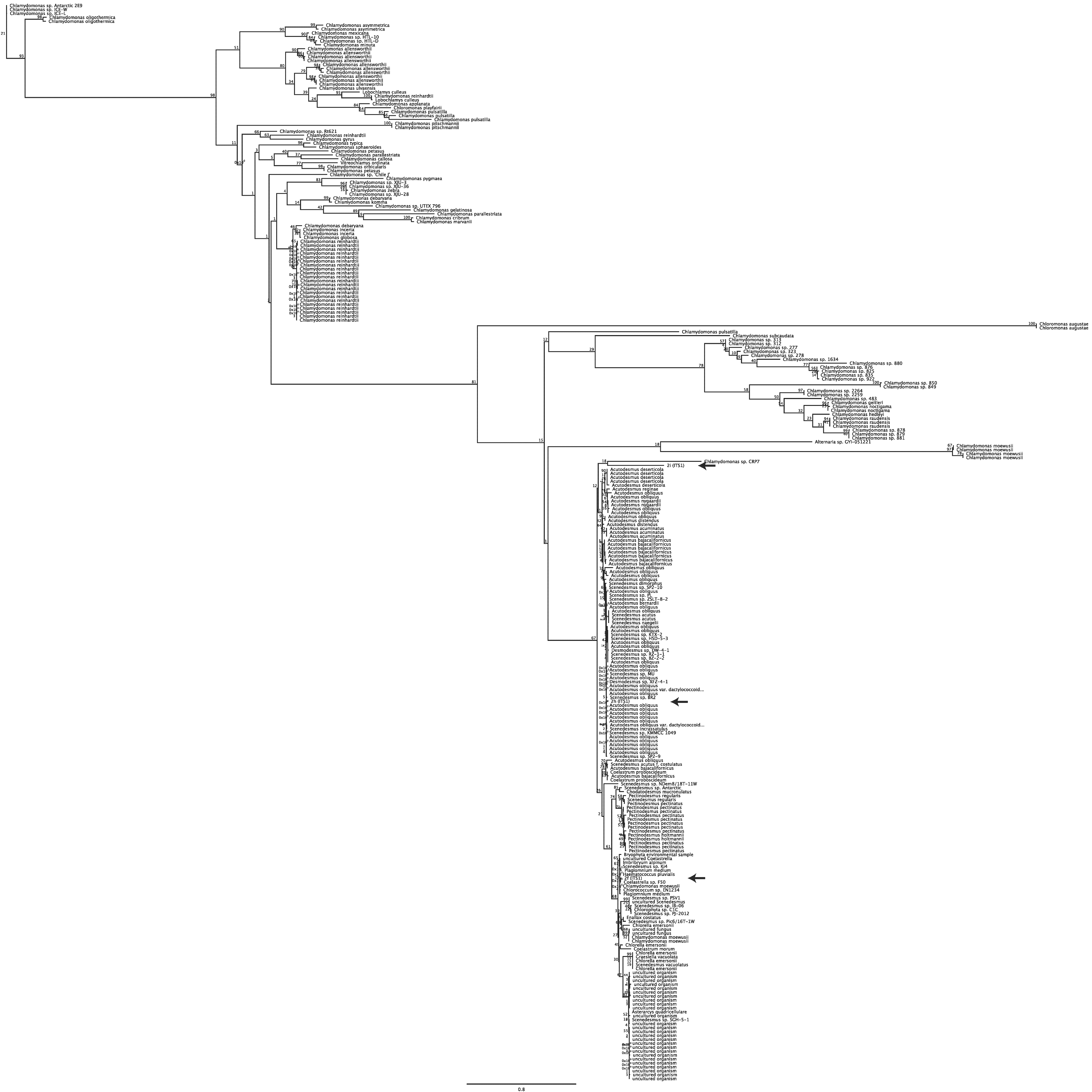


Figure S1. Phylogenetic tree of environmental isolates 2f, 2h and 2i based on ITS 1 ribosomal DNA sequence comparisons. Representative maximum likelihood phylogenies of ITS1 are provided for the three environmental isolates; ITS2 phylogenies are similar (Supplemental Fig S6). Bootstrap values, when available are indicated at each node.


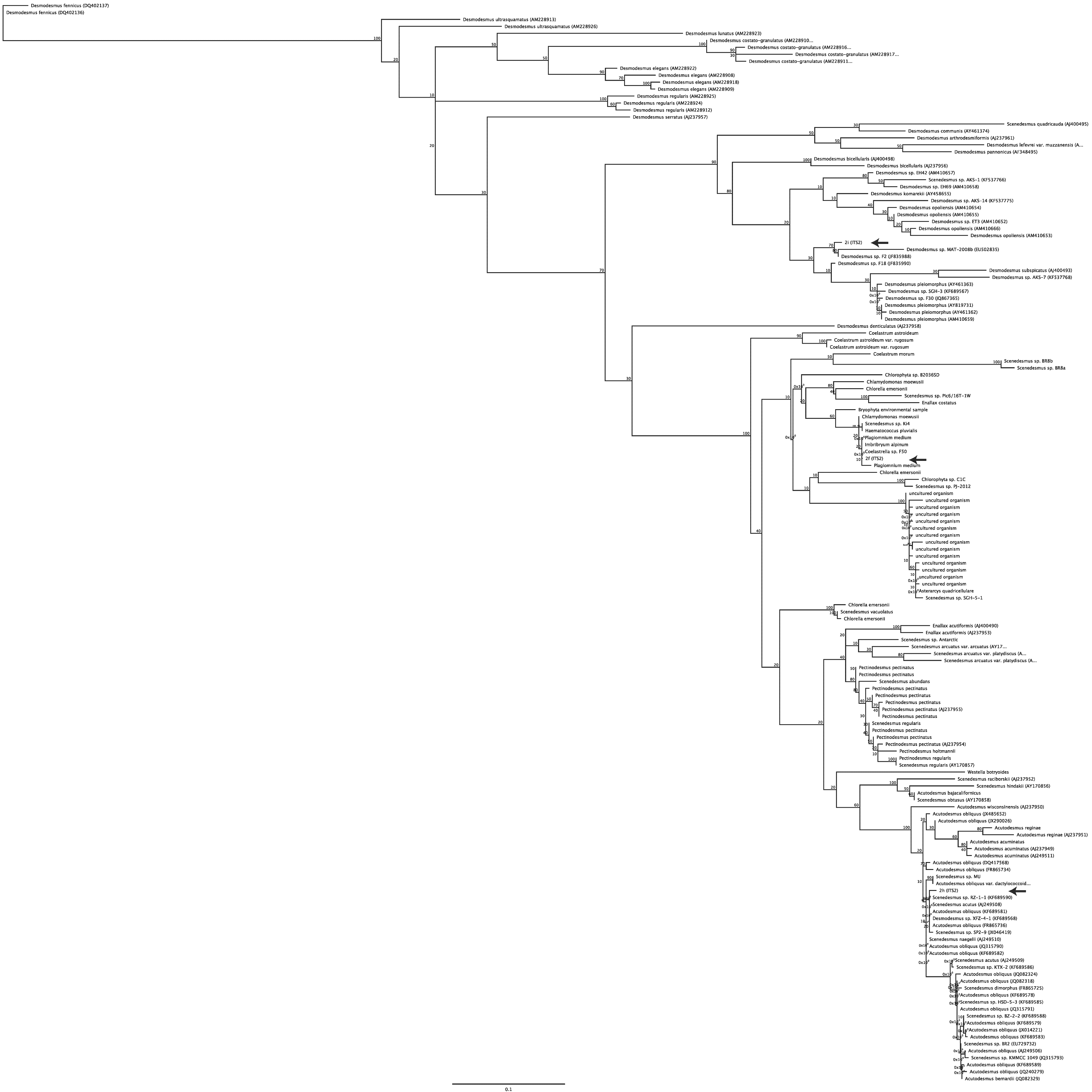


Figure S2. Phylogenetic tree of environmental isolates 2f, 2h and 2i based on ITS 2 ribosomal DNA sequence comparisons. Representative maximum likelihood phylogenies of ITS2 are provided for the three environmental isolates; ITS1 phylogenies are similar (Supplemental Fig S5). Bootstrap values, when available are indicated at each node.

Table S1. GenBank accession numbers for DNA sequences used for phylogenetic analyses.

| **GenBank ID** | **Figure** | **Strain Identification** | **ITS Sequence** |
| --- | --- | --- | --- |
| DQ417569 | Figure 9 | 2f | ITS1 |
| DQ417570 | Figure 9 | 2f | ITS1 |
| GQ375089 | Figure 9 | 2f | ITS1 |
| AY510465 | Figure 9 | 2f | ITS1 |
| AM419228 | Figure 9 | 2f | ITS1 |
| JQ082315 | Figure 9 | 2f | ITS1 |
| GQ375096 | Figure 9 | 2f | ITS1 |
| DQ417571 | Figure 9 | 2f | ITS1 |
| KC216055 | Figure 9 | 2f | ITS1 |
| KC216055 | Figure 9 | 2f | ITS1 |
| JX262262 | Figure 9 | 2f | ITS1 |
| JQ867368 | Figure 9 | 2f | ITS1 |
| FJ593899 | Figure 9 | 2f | ITS1 |
| FJ593899 | Figure 9 | 2f | ITS1 |
| AB762691 | Figure 9 | 2f | ITS1 |
| JX290025 | Figure 9 | 2f | ITS1 |
| JX046429 | Figure 9 | 2f | ITS1 |
| JX046429 | Figure 9 | 2f | ITS1 |
| FJ796893 | Figure 9 | 2f | ITS1 |
| FJ796893 | Figure 9 | 2f | ITS1 |
| AB762691 | Figure 9 | 2f | ITS1 |
| JQ867368 | Figure 9 | 2f | ITS1 |
| JX290025 | Figure 9 | 2f | ITS1 |
| JX262262 | Figure 9 | 2f | ITS1 |
| JQ082315 | Figure 9 | 2f | ITS2 |
| JQ240287 | Figure 9 | 2f | ITS2 |
| AM419228 | Figure 9 | 2f | ITS2 |
| AJ237953 | Figure 9 | 2f | ITS2 |
| AJ400490 | Figure 9 | 2f | ITS2 |
| AY170855 | Figure 9 | 2f | ITS2 |
| AJ400491 | Figure 9 | 2f | ITS2 |
| AY170854 | Figure 9 | 2f | ITS2 |
| GQ375089 | Figure 9 | 2f | ITS2 |
| JQ240288 | Figure 9 | 2f | ITS2 |
| JQ240289 | Figure 9 | 2f | ITS2 |
| JX290025 | Figure 9 | 2f | ITS2 |
| AB762691 | Figure 9 | 2f | ITS2 |
| JX046429 | Figure 9 | 2f | ITS2 |
| JQ867368 | Figure 9 | 2f | ITS2 |
| FJ796893 | Figure 9 | 2f | ITS2 |
| EU878192 | Figure 9 | 2f | ITS2 |
| FJ593899 | Figure 9 | 2f | ITS2 |
| EU878192 | Figure 9 | 2f | ITS2 |
| FJ593899 | Figure 9 | 2f | ITS2 |
| FJ796893 | Figure 9 | 2f | ITS2 |
| JQ867368 | Figure 9 | 2f | ITS2 |
| JX046429 | Figure 9 | 2f | ITS2 |
| AB762691 | Figure 9 | 2f | ITS2 |
| JX290025 | Figure 9 | 2f | ITS2 |
| JX485652 | Figure 9 | 2h | ITS1 |
| JX485652 | Figure 9 | 2h | ITS1 |
| AJ249511 | Figure 9 | 2h | ITS1 |
| JX290026 | Figure 9 | 2h | ITS1 |
| FR865725 | Figure 9 | 2h | ITS1 |
| JX046431 | Figure 9 | 2h | ITS1 |
| JX046431 | Figure 9 | 2h | ITS1 |
| KF689577 | Figure 9 | 2h | ITS1 |
| JX046428 | Figure 9 | 2h | ITS1 |
| KF689586 | Figure 9 | 2h | ITS1 |
| KF689568 | Figure 9 | 2h | ITS1 |
| EU729732 | Figure 9 | 2h | ITS1 |
| KF689589 | Figure 9 | 2h | ITS1 |
| KF689578 | Figure 9 | 2h | ITS1 |
| KF689588 | Figure 9 | 2h | ITS1 |
| KF689583 | Figure 9 | 2h | ITS1 |
| KF689562 | Figure 9 | 2h | ITS1 |
| JX014221 | Figure 9 | 2h | ITS1 |
| JX014221 | Figure 9 | 2h | ITS1 |
| KF689592 | Figure 9 | 2h | ITS1 |
| KF689580 | Figure 9 | 2h | ITS1 |
| KF689585 | Figure 9 | 2h | ITS1 |
| KF689590 | Figure 9 | 2h | ITS1 |
| KF689582 | Figure 9 | 2h | ITS1 |
| JX046427 | Figure 9 | 2h | ITS1 |
| KF689579 | Figure 9 | 2h | ITS1 |
| JQ082329 | Figure 9 | 2h | ITS1 |
| JQ082319 | Figure 9 | 2h | ITS1 |
| JQ082318 | Figure 9 | 2h | ITS1 |
| JQ082316 | Figure 9 | 2h | ITS1 |
| JQ082324 | Figure 9 | 2h | ITS1 |
| AJ249510 | Figure 9 | 2h | ITS1 |
| AJ249508 | Figure 9 | 2h | ITS1 |
| AJ249505 | Figure 9 | 2h | ITS1 |
| AJ249507 | Figure 9 | 2h | ITS1 |
| FR865715 | Figure 9 | 2h | ITS1 |
| FR865715 | Figure 9 | 2h | ITS1 |
| JX262261 | Figure 9 | 2h | ITS1 |
| JQ315793 | Figure 9 | 2h | ITS1 |
| JQ315791 | Figure 9 | 2h | ITS1 |
| JQ315790 | Figure 9 | 2h | ITS1 |
| FR865738 | Figure 9 | 2h | ITS1 |
| FR865737 | Figure 9 | 2h | ITS1 |
| FR865726 | Figure 9 | 2h | ITS1 |
| FR865736 | Figure 9 | 2h | ITS1 |
| AJ249506 | Figure 9 | 2h | ITS1 |
| FR865721 | Figure 9 | 2h | ITS1 |
| KF689581 | Figure 9 | 2h | ITS1 |
| KF689581 | Figure 9 | 2h | ITS1 |
| JX046419 | Figure 9 | 2h | ITS1 |
| FR865719 | Figure 9 | 2h | ITS1 |
| JX046427 | Figure 9 | 2h | ITS1 |
| KF689582 | Figure 9 | 2h | ITS1 |
| KF689577 | Figure 9 | 2h | ITS1 |
| KF689588 | Figure 9 | 2h | ITS1 |
| KF689562 | Figure 9 | 2h | ITS1 |
| KF689589 | Figure 9 | 2h | ITS1 |
| KF689578 | Figure 9 | 2h | ITS1 |
| KF689592 | Figure 9 | 2h | ITS1 |
| KF689586 | Figure 9 | 2h | ITS1 |
| KF689583 | Figure 9 | 2h | ITS1 |
| KF689585 | Figure 9 | 2h | ITS1 |
| KF689590 | Figure 9 | 2h | ITS1 |
| KF689579 | Figure 9 | 2h | ITS1 |
| KF689568 | Figure 9 | 2h | ITS1 |
| KF689580 | Figure 9 | 2h | ITS1 |
| JX290026 | Figure 9 | 2h | ITS1 |
| JX046428 | Figure 9 | 2h | ITS1 |
| JX046419 | Figure 9 | 2h | ITS1 |
| EU729732 | Figure 9 | 2h | ITS1 |
| JQ082329 | Figure 9 | 2h | ITS1 |
| JQ082319 | Figure 9 | 2h | ITS1 |
| JQ082318 | Figure 9 | 2h | ITS1 |
| JQ082316 | Figure 9 | 2h | ITS1 |
| JQ082324 | Figure 9 | 2h | ITS1 |
| JX262261 | Figure 9 | 2h | ITS1 |
| JQ315791 | Figure 9 | 2h | ITS1 |
| JQ315790 | Figure 9 | 2h | ITS1 |
| JQ315793 | Figure 9 | 2h | ITS1 |
| FR865726 | Figure 9 | 2h | ITS1 |
| FR865738 | Figure 9 | 2h | ITS1 |
| FR865737 | Figure 9 | 2h | ITS1 |
| FR865736 | Figure 9 | 2h | ITS1 |
| FR865721 | Figure 9 | 2h | ITS1 |
| FR865719 | Figure 9 | 2h | ITS1 |
| FR865725 | Figure 9 | 2h | ITS1 |
| AJ237949 | Figure 9 | 2h | ITS2 |
| AJ249511 | Figure 9 | 2h | ITS2 |
| JX290026 | Figure 9 | 2h | ITS2 |
| JX485652 | Figure 9 | 2h | ITS2 |
| DQ417568 | Figure 9 | 2h | ITS2 |
| FR865734 | Figure 9 | 2h | ITS2 |
| FR865726 | Figure 9 | 2h | ITS2 |
| JQ240279 | Figure 9 | 2h | ITS2 |
| AJ249510 | Figure 9 | 2h | ITS2 |
| JQ315793 | Figure 9 | 2h | ITS2 |
| AJ249506 | Figure 9 | 2h | ITS2 |
| JQ082329 | Figure 9 | 2h | ITS2 |
| FR865715 | Figure 9 | 2h | ITS2 |
| JQ082319 | Figure 9 | 2h | ITS2 |
| FR865738 | Figure 9 | 2h | ITS2 |
| EU729732 | Figure 9 | 2h | ITS2 |
| JQ315790 | Figure 9 | 2h | ITS2 |
| KF689582 | Figure 9 | 2h | ITS2 |
| JX046419 | Figure 9 | 2h | ITS2 |
| FR865737 | Figure 9 | 2h | ITS2 |
| KF689568 | Figure 9 | 2h | ITS2 |
| JX046431 | Figure 9 | 2h | ITS2 |
| AJ249508 | Figure 9 | 2h | ITS2 |
| KF689590 | Figure 9 | 2h | ITS2 |
| KF689581 | Figure 9 | 2h | ITS2 |
| FR865736 | Figure 9 | 2h | ITS2 |
| AJ249509 | Figure 9 | 2h | ITS2 |
| KF689586 | Figure 9 | 2h | ITS2 |
| KF689589 | Figure 9 | 2h | ITS2 |
| KF689583 | Figure 9 | 2h | ITS2 |
| KF689579 | Figure 9 | 2h | ITS2 |
| AJ249505 | Figure 9 | 2h | ITS2 |
| KF689588 | Figure 9 | 2h | ITS2 |
| JX014221 | Figure 9 | 2h | ITS2 |
| KF689578 | Figure 9 | 2h | ITS2 |
| FR865725 | Figure 9 | 2h | ITS2 |
| KF689585 | Figure 9 | 2h | ITS2 |
| JQ082318 | Figure 9 | 2h | ITS2 |
| AJ249507 | Figure 9 | 2h | ITS2 |
| JQ315791 | Figure 9 | 2h | ITS2 |
| JQ082324 | Figure 9 | 2h | ITS2 |
| JQ240279 | Figure 9 | 2h | ITS2 |
| AJ249510 | Figure 9 | 2h | ITS2 |
| AJ249508 | Figure 9 | 2h | ITS2 |
| AJ249509 | Figure 9 | 2h | ITS2 |
| JQ082329 | Figure 9 | 2h | ITS2 |
| JQ082319 | Figure 9 | 2h | ITS2 |
| EU729732 | Figure 9 | 2h | ITS2 |
| JQ082318 | Figure 9 | 2h | ITS2 |
| JQ082324 | Figure 9 | 2h | ITS2 |
| KF689581 | Figure 9 | 2h | ITS2 |
| JX046431 | Figure 9 | 2h | ITS2 |
| KF689588 | Figure 9 | 2h | ITS2 |
| KF689589 | Figure 9 | 2h | ITS2 |
| KF689578 | Figure 9 | 2h | ITS2 |
| KF689590 | Figure 9 | 2h | ITS2 |
| KF689583 | Figure 9 | 2h | ITS2 |
| KF689579 | Figure 9 | 2h | ITS2 |
| KF689585 | Figure 9 | 2h | ITS2 |
| KF689568 | Figure 9 | 2h | ITS2 |
| KF689586 | Figure 9 | 2h | ITS2 |
| KF689582 | Figure 9 | 2h | ITS2 |
| JX014221 | Figure 9 | 2h | ITS2 |
| JX485652 | Figure 9 | 2h | ITS2 |
| DQ417568 | Figure 9 | 2h | ITS2 |
| FR865715 | Figure 9 | 2h | ITS2 |
| JX290026 | Figure 9 | 2h | ITS2 |
| JX046419 | Figure 9 | 2h | ITS2 |
| JQ315791 | Figure 9 | 2h | ITS2 |
| JQ315790 | Figure 9 | 2h | ITS2 |
| JQ315793 | Figure 9 | 2h | ITS2 |
| FR865726 | Figure 9 | 2h | ITS2 |
| FR865738 | Figure 9 | 2h | ITS2 |
| FR865737 | Figure 9 | 2h | ITS2 |
| FR865736 | Figure 9 | 2h | ITS2 |
| FR865734 | Figure 9 | 2h | ITS2 |
| FR865725 | Figure 9 | 2h | ITS2 |
| JQ867365 | Figure 9 | 2i | ITS1 |
| JF835990 | Figure 9 | 2i | ITS1 |
| JF835987 | Figure 9 | 2i | ITS1 |
| JF835988 | Figure 9 | 2i | ITS1 |
| EU502835 | Figure 9 | 2i | ITS1 |
| JF835990 | Figure 9 | 2i | ITS1 |
| JF835987 | Figure 9 | 2i | ITS1 |
| JF835988 | Figure 9 | 2i | ITS1 |
| JQ867365 | Figure 9 | 2i | ITS1 |
| KF689567 | Figure 9 | 2i | ITS1 |
| KC216052 | Figure 9 | 2i | ITS1 |
| KC216052 | Figure 9 | 2i | ITS1 |
| KF537768 | Figure 9 | 2i | ITS1 |
| KF537768 | Figure 9 | 2i | ITS1 |
| KC215958 | Figure 9 | 2i | ITS1 |
| KC215958 | Figure 9 | 2i | ITS1 |
| KC216006 | Figure 9 | 2i | ITS1 |
| KC216006 | Figure 9 | 2i | ITS1 |
| EU502835 | Figure 9 | 2i | ITS1 |
| KF689567 | Figure 9 | 2i | ITS1 |
| AM410666 | Figure 9 | 2i | ITS2 |
| KF537775 | Figure 9 | 2i | ITS2 |
| AM410654 | Figure 9 | 2i | ITS2 |
| AM410653 | Figure 9 | 2i | ITS2 |
| AM410655 | Figure 9 | 2i | ITS2 |
| AM410652 | Figure 9 | 2i | ITS2 |
| AY458655 | Figure 9 | 2i | ITS2 |
| AM410657 | Figure 9 | 2i | ITS2 |
| KF537766 | Figure 9 | 2i | ITS2 |
| AM410658 | Figure 9 | 2i | ITS2 |
| KF537768 | Figure 9 | 2i | ITS2 |
| AY461362 | Figure 9 | 2i | ITS2 |
| JF835990 | Figure 9 | 2i | ITS2 |
| JF835987 | Figure 9 | 2i | ITS2 |
| JF835988 | Figure 9 | 2i | ITS2 |
| AY461363 | Figure 9 | 2i | ITS2 |
| KF689567 | Figure 9 | 2i | ITS2 |
| AM410659 | Figure 9 | 2i | ITS2 |
| AY819731 | Figure 9 | 2i | ITS2 |
| JQ867365 | Figure 9 | 2i | ITS2 |
| EU502835 | Figure 9 | 2i | ITS2 |
| AY461362 | Figure 9 | 2i | ITS2 |
| AY819731 | Figure 9 | 2i | ITS2 |
| AY461363 | Figure 9 | 2i | ITS2 |
| AM410666 | Figure 9 | 2i | ITS2 |
| AM410654 | Figure 9 | 2i | ITS2 |
| AM410653 | Figure 9 | 2i | ITS2 |
| AM410655 | Figure 9 | 2i | ITS2 |
| AM410652 | Figure 9 | 2i | ITS2 |
| AM410657 | Figure 9 | 2i | ITS2 |
| AM410658 | Figure 9 | 2i | ITS2 |
| AM410659 | Figure 9 | 2i | ITS2 |
| JF835987 | Figure 9 | 2i | ITS2 |
| JF835988 | Figure 9 | 2i | ITS2 |
| JF835990 | Figure 9 | 2i | ITS2 |
| JQ867365 | Figure 9 | 2i | ITS2 |
| KF689567 | Figure 9 | 2i | ITS2 |
| EU502835 | Figure 9 | 2i | ITS2 |
| KF537768 | Figure 9 | 2i | ITS2 |
| KF537766 | Figure 9 | 2i | ITS2 |
| KF537775 | Figure 9 | 2i | ITS2 |
| AJ249511 | Supplemental Figure S5 | All | ITS1 |
| AJ249511 | Supplemental Figure S5 | All | ITS1 |
| AJ249506 | Supplemental Figure S5 | All | ITS1 |
| AJ249508 | Supplemental Figure S5 | All | ITS1 |
| AJ249509 | Supplemental Figure S5 | All | ITS1 |
| AJ249510 | Supplemental Figure S5 | All | ITS1 |
| AM419228 | Supplemental Figure S5 | All | ITS1 |
| AB001487 | Supplemental Figure S5 | All | ITS1 |
| AY731084 | Supplemental Figure S5 | All | ITS1 |
| AY731085 | Supplemental Figure S5 | All | ITS1 |
| HQ404879 | Supplemental Figure S5 | All | ITS1 |
| HQ404898 | Supplemental Figure S5 | All | ITS1 |
| AJ749616 | Supplemental Figure S5 | All | ITS1 |
| CAU66944 | Supplemental Figure S5 | All | ITS1 |
| CMU66953 | Supplemental Figure S5 | All | ITS1 |
| FJ888523 | Supplemental Figure S5 | All | ITS1 |
| FJ888522 | Supplemental Figure S5 | All | ITS1 |
| AJ749617 | Supplemental Figure S5 | All | ITS1 |
| AF326855 | Supplemental Figure S5 | All | ITS1 |
| AF326850 | Supplemental Figure S5 | All | ITS1 |
| AF326849 | Supplemental Figure S5 | All | ITS1 |
| AF326854 | Supplemental Figure S5 | All | ITS1 |
| AF033285 | Supplemental Figure S5 | All | ITS1 |
| CAU66943 | Supplemental Figure S5 | All | ITS1 |
| AF033284 | Supplemental Figure S5 | All | ITS1 |
| AF326851 | Supplemental Figure S5 | All | ITS1 |
| AF326852 | Supplemental Figure S5 | All | ITS1 |
| AF326853 | Supplemental Figure S5 | All | ITS1 |
| AJ749612 | Supplemental Figure S5 | All | ITS1 |
| CCU66946 | Supplemental Figure S5 | All | ITS1 |
| AF033292 | Supplemental Figure S5 | All | ITS1 |
| AF033293 | Supplemental Figure S5 | All | ITS1 |
| DQ377088 | Supplemental Figure S5 | All | ITS1 |
| AF156602 | Supplemental Figure S5 | All | ITS1 |
| AB001483 | Supplemental Figure S5 | All | ITS1 |
| AB001484 | Supplemental Figure S5 | All | ITS1 |
| AB001918 | Supplemental Figure S5 | All | ITS1 |
| CPITITS1 1 | Supplemental Figure S5 | All | ITS1 |
| CPITITS1 2 | Supplemental Figure S5 | All | ITS1 |
| AF033277 | Supplemental Figure S5 | All | ITS1 |
| AF033286 | Supplemental Figure S5 | All | ITS1 |
| AJ749618 | Supplemental Figure S5 | All | ITS1 |
| AJ749622 | Supplemental Figure S5 | All | ITS1 |
| CLU66952 | Supplemental Figure S5 | All | ITS1 |
| AJ749615 | Supplemental Figure S5 | All | ITS1 |
| AJ749613 | Supplemental Figure S5 | All | ITS1 |
| CCU66945 | Supplemental Figure S5 | All | ITS1 |
| AJ749611 | Supplemental Figure S5 | All | ITS1 |
| AB511844 | Supplemental Figure S5 | All | ITS1 |
| AJ749614 | Supplemental Figure S5 | All | ITS1 |
| AF033295 | Supplemental Figure S5 | All | ITS1 |
| AJ749619 | Supplemental Figure S5 | All | ITS1 |
| FJ589633 | Supplemental Figure S5 | All | ITS1 |
| FJ572059 | Supplemental Figure S5 | All | ITS1 |
| AF033294 | Supplemental Figure S5 | All | ITS1 |
| FJ589634 | Supplemental Figure S5 | All | ITS1 |
| AJ749627 | Supplemental Figure S5 | All | ITS1 |
| CKU66951 | Supplemental Figure S5 | All | ITS1 |
| AJ749621 | Supplemental Figure S5 | All | ITS1 |
| CGU66948 | Supplemental Figure S5 | All | ITS1 |
| AJ749623 | Supplemental Figure S5 | All | ITS1 |
| AF033281 | Supplemental Figure S5 | All | ITS1 |
| AJ749620 | Supplemental Figure S5 | All | ITS1 |
| AJ749626 | Supplemental Figure S5 | All | ITS1 |
| AJ749625 | Supplemental Figure S5 | All | ITS1 |
| CIU66950 | Supplemental Figure S5 | All | ITS1 |
| AB511843 | Supplemental Figure S5 | All | ITS1 |
| AJ749624 | Supplemental Figure S5 | All | ITS1 |
| AF033291 | Supplemental Figure S5 | All | ITS1 |
| AF033290 | Supplemental Figure S5 | All | ITS1 |
| AJ749635 | Supplemental Figure S5 | All | ITS1 |
| AF033288 | Supplemental Figure S5 | All | ITS1 |
| AF156601 | Supplemental Figure S5 | All | ITS1 |
| AF033289 | Supplemental Figure S5 | All | ITS1 |
| CRU66954 | Supplemental Figure S5 | All | ITS1 |
| AJ749633 | Supplemental Figure S5 | All | ITS1 |
| AJ749634 | Supplemental Figure S5 | All | ITS1 |
| AJ749636 | Supplemental Figure S5 | All | ITS1 |
| AJ749637 | Supplemental Figure S5 | All | ITS1 |
| AJ749638 | Supplemental Figure S5 | All | ITS1 |
| AJ749631 | Supplemental Figure S5 | All | ITS1 |
| AJ749629 | Supplemental Figure S5 | All | ITS1 |
| AB511842 | Supplemental Figure S5 | All | ITS1 |
| AJ749632 | Supplemental Figure S5 | All | ITS1 |
| AJ749630 | Supplemental Figure S5 | All | ITS1 |
| AJ749628 | Supplemental Figure S5 | All | ITS1 |
| AB001537 | Supplemental Figure S5 | All | ITS1 |
| AF033287 | Supplemental Figure S5 | All | ITS1 |
| HQ404869 | Supplemental Figure S5 | All | ITS1 |
| HQ404886 | Supplemental Figure S5 | All | ITS1 |
| AB001917 | Supplemental Figure S5 | All | ITS1 |
| HQ404896 | Supplemental Figure S5 | All | ITS1 |
| AJ297812 | Supplemental Figure S5 | All | ITS1 |
| AJ297813 | Supplemental Figure S5 | All | ITS1 |
| AJ297811 | Supplemental Figure S5 | All | ITS1 |
| AJ297809 | Supplemental Figure S5 | All | ITS1 |
| AJ297810 | Supplemental Figure S5 | All | ITS1 |
| AJ297814 | Supplemental Figure S5 | All | ITS1 |
| AJ297798 | Supplemental Figure S5 | All | ITS1 |
| AJ297803 | Supplemental Figure S5 | All | ITS1 |
| AJ297802 | Supplemental Figure S5 | All | ITS1 |
| AJ297804 | Supplemental Figure S5 | All | ITS1 |
| AJ297805 | Supplemental Figure S5 | All | ITS1 |
| AJ297795 | Supplemental Figure S5 | All | ITS1 |
| AJ297796 | Supplemental Figure S5 | All | ITS1 |
| AJ297806 | Supplemental Figure S5 | All | ITS1 |
| AJ297807 | Supplemental Figure S5 | All | ITS1 |
| AJ297808 | Supplemental Figure S5 | All | ITS1 |
| CGU66947 | Supplemental Figure S5 | All | ITS1 |
| AF033282 | Supplemental Figure S5 | All | ITS1 |
| AF033283 | Supplemental Figure S5 | All | ITS1 |
| AJ297797 | Supplemental Figure S5 | All | ITS1 |
| AJ781315 | Supplemental Figure S5 | All | ITS1 |
| AJ781314 | Supplemental Figure S5 | All | ITS1 |
| AJ781316 | Supplemental Figure S5 | All | ITS1 |
| AJ297800 | Supplemental Figure S5 | All | ITS1 |
| AJ297799 | Supplemental Figure S5 | All | ITS1 |
| AJ297801 | Supplemental Figure S5 | All | ITS1 |
| FJ627005 | Supplemental Figure S5 | All | ITS1 |
| CHLMITS1 2 | Supplemental Figure S5 | All | ITS1 |
| CHLMITS1 1 | Supplemental Figure S5 | All | ITS1 |
| CEITS1 2 | Supplemental Figure S5 | All | ITS1 |
| CEITS1 1 | Supplemental Figure S5 | All | ITS1 |
| JQ408690 | Supplemental Figure S5 | All | ITS1 |
| AY510474 | Supplemental Figure S5 | All | ITS1 |
| AY510470 | Supplemental Figure S5 | All | ITS1 |
| AY510471 | Supplemental Figure S5 | All | ITS1 |
| AY510472 | Supplemental Figure S5 | All | ITS1 |
| AY510473 | Supplemental Figure S5 | All | ITS1 |
| JQ082326 | Supplemental Figure S5 | All | ITS1 |
| KC792552 | Supplemental Figure S5 | All | ITS1 |
| JX041598 | Supplemental Figure S5 | All | ITS1 |
| JQ082320 | Supplemental Figure S5 | All | ITS1 |
| JQ082325 | Supplemental Figure S5 | All | ITS1 |
| JX519262 | Supplemental Figure S5 | All | ITS1 |
| FR865729 | Supplemental Figure S5 | All | ITS1 |
| FR865731 | Supplemental Figure S5 | All | ITS1 |
| JQ082322 | Supplemental Figure S5 | All | ITS1 |
| JQ082313 | Supplemental Figure S5 | All | ITS1 |
| JQ082312 | Supplemental Figure S5 | All | ITS1 |
| JQ082330 | Supplemental Figure S5 | All | ITS1 |
| HQ246450 | Supplemental Figure S5 | All | ITS1 |
| HQ246449 | Supplemental Figure S5 | All | ITS1 |
| HQ246448 | Supplemental Figure S5 | All | ITS1 |
| AY510469 | Supplemental Figure S5 | All | ITS1 |
| AY510468 | Supplemental Figure S5 | All | ITS1 |
| AY510467 | Supplemental Figure S5 | All | ITS1 |
| HQ246447 | Supplemental Figure S5 | All | ITS1 |
| JX485652 | Supplemental Figure S5 | All | ITS1 |
| DQ417568 | Supplemental Figure S5 | All | ITS1 |
| FR865734 | Supplemental Figure S5 | All | ITS1 |
| JX290026 | Supplemental Figure S5 | All | ITS1 |
| FR865725 | Supplemental Figure S5 | All | ITS1 |
| JX046431 | Supplemental Figure S5 | All | ITS1 |
| KF689581 | Supplemental Figure S5 | All | ITS1 |
| JX046427 | Supplemental Figure S5 | All | ITS1 |
| KF689592 | Supplemental Figure S5 | All | ITS1 |
| JQ082329 | Supplemental Figure S5 | All | ITS1 |
| JQ082324 | Supplemental Figure S5 | All | ITS1 |
| KF689582 | Supplemental Figure S5 | All | ITS1 |
| KF689579 | Supplemental Figure S5 | All | ITS1 |
| KF689586 | Supplemental Figure S5 | All | ITS1 |
| KF689585 | Supplemental Figure S5 | All | ITS1 |
| KF689589 | Supplemental Figure S5 | All | ITS1 |
| KF689583 | Supplemental Figure S5 | All | ITS1 |
| KF689562 | Supplemental Figure S5 | All | ITS1 |
| KF689590 | Supplemental Figure S5 | All | ITS1 |
| KF689588 | Supplemental Figure S5 | All | ITS1 |
| KF689578 | Supplemental Figure S5 | All | ITS1 |
| KF689580 | Supplemental Figure S5 | All | ITS1 |
| JQ082319 | Supplemental Figure S5 | All | ITS1 |
| JX046428 | Supplemental Figure S5 | All | ITS1 |
| KF689577 | Supplemental Figure S5 | All | ITS1 |
| KF689568 | Supplemental Figure S5 | All | ITS1 |
| JQ082318 | Supplemental Figure S5 | All | ITS1 |
| JQ082316 | Supplemental Figure S5 | All | ITS1 |
| FR865715 | Supplemental Figure S5 | All | ITS1 |
| EU729732 | Supplemental Figure S5 | All | ITS1 |
| FR865726 | Supplemental Figure S5 | All | ITS1 |
| JX014221 | Supplemental Figure S5 | All | ITS1 |
| FR865736 | Supplemental Figure S5 | All | ITS1 |
| FR865738 | Supplemental Figure S5 | All | ITS1 |
| FR865721 | Supplemental Figure S5 | All | ITS1 |
| JQ082317 | Supplemental Figure S5 | All | ITS1 |
| FR865722 | Supplemental Figure S5 | All | ITS1 |
| JQ315793 | Supplemental Figure S5 | All | ITS1 |
| FR865737 | Supplemental Figure S5 | All | ITS1 |
| FR865719 | Supplemental Figure S5 | All | ITS1 |
| JX262261 | Supplemental Figure S5 | All | ITS1 |
| JQ315790 | Supplemental Figure S5 | All | ITS1 |
| JQ315791 | Supplemental Figure S5 | All | ITS1 |
| JX046419 | Supplemental Figure S5 | All | ITS1 |
| JQ782745 | Supplemental Figure S5 | All | ITS1 |
| JQ315789 | Supplemental Figure S5 | All | ITS1 |
| JX456466 | Supplemental Figure S5 | All | ITS1 |
| JQ898144 | Supplemental Figure S5 | All | ITS1 |
| JQ782744 | Supplemental Figure S5 | All | ITS1 |
| JX485653 | Supplemental Figure S5 | All | ITS1 |
| DQ417550 | Supplemental Figure S5 | All | ITS1 |
| JQ082315 | Supplemental Figure S5 | All | ITS1 |
| JQ082323 | Supplemental Figure S5 | All | ITS1 |
| FR865732 | Supplemental Figure S5 | All | ITS1 |
| JQ082328 | Supplemental Figure S5 | All | ITS1 |
| FR865730 | Supplemental Figure S5 | All | ITS1 |
| JQ082321 | Supplemental Figure S5 | All | ITS1 |
| FR865723 | Supplemental Figure S5 | All | ITS1 |
| JQ082314 | Supplemental Figure S5 | All | ITS1 |
| JQ082327 | Supplemental Figure S5 | All | ITS1 |
| JQ082331 | Supplemental Figure S5 | All | ITS1 |
| JN703737 | Supplemental Figure S5 | All | ITS1 |
| JQ082334 | Supplemental Figure S5 | All | ITS1 |
| JQ082335 | Supplemental Figure S5 | All | ITS1 |
| FR865735 | Supplemental Figure S5 | All | ITS1 |
| JQ082332 | Supplemental Figure S5 | All | ITS1 |
| FR865733 | Supplemental Figure S5 | All | ITS1 |
| JF737776 | Supplemental Figure S5 | All | ITS1 |
| KC216055 | Supplemental Figure S5 | All | ITS1 |
| FJ593899 | Supplemental Figure S5 | All | ITS1 |
| AB762691 | Supplemental Figure S5 | All | ITS1 |
| EU878192 | Supplemental Figure S5 | All | ITS1 |
| JX046429 | Supplemental Figure S5 | All | ITS1 |
| JQ867368 | Supplemental Figure S5 | All | ITS1 |
| JX290025 | Supplemental Figure S5 | All | ITS1 |
| JX262262 | Supplemental Figure S5 | All | ITS1 |
| FJ796893 | Supplemental Figure S5 | All | ITS1 |
| JX519261 | Supplemental Figure S5 | All | ITS1 |
| KC216053 | Supplemental Figure S5 | All | ITS1 |
| JQ782746 | Supplemental Figure S5 | All | ITS1 |
| JX046434 | Supplemental Figure S5 | All | ITS1 |
| JN832676 | Supplemental Figure S5 | All | ITS1 |
| AB762692 | Supplemental Figure S5 | All | ITS1 |
| DQ417571 | Supplemental Figure S5 | All | ITS1 |
| FR865674 | Supplemental Figure S5 | All | ITS1 |
| GU461406 | Supplemental Figure S5 | All | ITS1 |
| GU461407 | Supplemental Figure S5 | All | ITS1 |
| FR865601 | Supplemental Figure S5 | All | ITS1 |
| FR865599 | Supplemental Figure S5 | All | ITS1 |
| JX456465 | Supplemental Figure S5 | All | ITS1 |
| GQ375096 | Supplemental Figure S5 | All | ITS1 |
| FR865687 | Supplemental Figure S5 | All | ITS1 |
| FR865685 | Supplemental Figure S5 | All | ITS1 |
| FR865661 | Supplemental Figure S5 | All | ITS1 |
| AB762693 | Supplemental Figure S5 | All | ITS1 |
| FR865657 | Supplemental Figure S5 | All | ITS1 |
| JN660610 | Supplemental Figure S5 | All | ITS1 |
| JN660609 | Supplemental Figure S5 | All | ITS1 |
| JN660711 | Supplemental Figure S5 | All | ITS1 |
| JN660643 | Supplemental Figure S5 | All | ITS1 |
| JN660716 | Supplemental Figure S5 | All | ITS1 |
| JN660633 | Supplemental Figure S5 | All | ITS1 |
| JN660618 | Supplemental Figure S5 | All | ITS1 |
| JN660628 | Supplemental Figure S5 | All | ITS1 |
| JN660644 | Supplemental Figure S5 | All | ITS1 |
| JN660606 | Supplemental Figure S5 | All | ITS1 |
| JQ043184 | Supplemental Figure S5 | All | ITS1 |
| JN660664 | Supplemental Figure S5 | All | ITS1 |
| KF689591 | Supplemental Figure S5 | All | ITS1 |
| JN660625 | Supplemental Figure S5 | All | ITS1 |
| JN660670 | Supplemental Figure S5 | All | ITS1 |
| JN660668 | Supplemental Figure S5 | All | ITS1 |
| JN660626 | Supplemental Figure S5 | All | ITS1 |
| JN660604 | Supplemental Figure S5 | All | ITS1 |
| JN660631 | Supplemental Figure S5 | All | ITS1 |
| JN660612 | Supplemental Figure S5 | All | ITS1 |
| JN660602 | Supplemental Figure S5 | All | ITS1 |
| JN660624 | Supplemental Figure S5 | All | ITS1 |
| JN660640 | Supplemental Figure S5 | All | ITS1 |
| JN660616 | Supplemental Figure S5 | All | ITS1 |
| JN660601 | Supplemental Figure S5 | All | ITS1 |
| JN660667 | Supplemental Figure S5 | All | ITS1 |
| JN660779 | Supplemental Figure S5 | All | ITS1 |
| JN660627 | Supplemental Figure S5 | All | ITS1 |
| DQ402137 | Supplemental Figure S6 | All | ITS2 |
| DQ402136 | Supplemental Figure S6 | All | ITS2 |
| AM228913 | Supplemental Figure S6 | All | ITS2 |
| AM228926 | Supplemental Figure S6 | All | ITS2 |
| AM228923 | Supplemental Figure S6 | All | ITS2 |
| AM228910 | Supplemental Figure S6 | All | ITS2 |
| AM228916 | Supplemental Figure S6 | All | ITS2 |
| AM228917 | Supplemental Figure S6 | All | ITS2 |
| AM228911 | Supplemental Figure S6 | All | ITS2 |
| AM228922 | Supplemental Figure S6 | All | ITS2 |
| AM228908 | Supplemental Figure S6 | All | ITS2 |
| AM228918 | Supplemental Figure S6 | All | ITS2 |
| AM228909 | Supplemental Figure S6 | All | ITS2 |
| AM228925 | Supplemental Figure S6 | All | ITS2 |
| AM228924 | Supplemental Figure S6 | All | ITS2 |
| AM228912 | Supplemental Figure S6 | All | ITS2 |
| AJ237957 | Supplemental Figure S6 | All | ITS2 |
| AJ400495 | Supplemental Figure S6 | All | ITS2 |
| AY461374 | Supplemental Figure S6 | All | ITS2 |
| AJ237961 | Supplemental Figure S6 | All | ITS2 |
| AJ237959 | Supplemental Figure S6 | All | ITS2 |
| AF348495 | Supplemental Figure S6 | All | ITS2 |
| AJ400498 | Supplemental Figure S6 | All | ITS2 |
| AJ237956 | Supplemental Figure S6 | All | ITS2 |
| AM410657 | Supplemental Figure S6 | All | ITS2 |
| KF537766 | Supplemental Figure S6 | All | ITS2 |
| AM410658 | Supplemental Figure S6 | All | ITS2 |
| AY458655 | Supplemental Figure S6 | All | ITS2 |
| KF537775 | Supplemental Figure S6 | All | ITS2 |
| AM410654 | Supplemental Figure S6 | All | ITS2 |
| AM410655 | Supplemental Figure S6 | All | ITS2 |
| AM410652 | Supplemental Figure S6 | All | ITS2 |
| AM410666 | Supplemental Figure S6 | All | ITS2 |
| AM410653 | Supplemental Figure S6 | All | ITS2 |
| EU502835 | Supplemental Figure S6 | All | ITS2 |
| JF835988 | Supplemental Figure S6 | All | ITS2 |
| JF835990 | Supplemental Figure S6 | All | ITS2 |
| AJ400493 | Supplemental Figure S6 | All | ITS2 |
| KF537768 | Supplemental Figure S6 | All | ITS2 |
| AY461363 | Supplemental Figure S6 | All | ITS2 |
| KF689567 | Supplemental Figure S6 | All | ITS2 |
| JQ867365 | Supplemental Figure S6 | All | ITS2 |
| AY819731 | Supplemental Figure S6 | All | ITS2 |
| AY461362 | Supplemental Figure S6 | All | ITS2 |
| AM410659 | Supplemental Figure S6 | All | ITS2 |
| AJ237958 | Supplemental Figure S6 | All | ITS2 |
| GQ375089 | Supplemental Figure S6 | All | ITS2 |
| GQ375093 | Supplemental Figure S6 | All | ITS2 |
| GQ375092 | Supplemental Figure S6 | All | ITS2 |
| GQ375096 | Supplemental Figure S6 | All | ITS2 |
| EU729730 | Supplemental Figure S6 | All | ITS2 |
| EU729729 | Supplemental Figure S6 | All | ITS2 |
| JQ615588 | Supplemental Figure S6 | All | ITS2 |
| FR865601 | Supplemental Figure S6 | All | ITS2 |
| FR865674 | Supplemental Figure S6 | All | ITS2 |
| DQ417571 | Supplemental Figure S6 | All | ITS2 |
| AB762692 | Supplemental Figure S6 | All | ITS2 |
| JF737776 | Supplemental Figure S6 | All | ITS2 |
| JX290025 | Supplemental Figure S6 | All | ITS2 |
| AB762691 | Supplemental Figure S6 | All | ITS2 |
| JX046429 | Supplemental Figure S6 | All | ITS2 |
| EU878192 | Supplemental Figure S6 | All | ITS2 |
| FJ593899 | Supplemental Figure S6 | All | ITS2 |
| JQ867368 | Supplemental Figure S6 | All | ITS2 |
| FJ796893 | Supplemental Figure S6 | All | ITS2 |
| JX456465 | Supplemental Figure S6 | All | ITS2 |
| JX046434 | Supplemental Figure S6 | All | ITS2 |
| JN832676 | Supplemental Figure S6 | All | ITS2 |
| JN660607 | Supplemental Figure S6 | All | ITS2 |
| JN660613 | Supplemental Figure S6 | All | ITS2 |
| JN660630 | Supplemental Figure S6 | All | ITS2 |
| JN660628 | Supplemental Figure S6 | All | ITS2 |
| JN660611 | Supplemental Figure S6 | All | ITS2 |
| JN660714 | Supplemental Figure S6 | All | ITS2 |
| JN660713 | Supplemental Figure S6 | All | ITS2 |
| JN660715 | Supplemental Figure S6 | All | ITS2 |
| JN660614 | Supplemental Figure S6 | All | ITS2 |
| JN660636 | Supplemental Figure S6 | All | ITS2 |
| JN660673 | Supplemental Figure S6 | All | ITS2 |
| JN660603 | Supplemental Figure S6 | All | ITS2 |
| JN660667 | Supplemental Figure S6 | All | ITS2 |
| JN660765 | Supplemental Figure S6 | All | ITS2 |
| JQ043184 | Supplemental Figure S6 | All | ITS2 |
| KF689591 | Supplemental Figure S6 | All | ITS2 |
| FR865687 | Supplemental Figure S6 | All | ITS2 |
| AB762693 | Supplemental Figure S6 | All | ITS2 |
| FR865661 | Supplemental Figure S6 | All | ITS2 |
| AJ400490 | Supplemental Figure S6 | All | ITS2 |
| AJ237953 | Supplemental Figure S6 | All | ITS2 |
| AM419228 | Supplemental Figure S6 | All | ITS2 |
| AY170854 | Supplemental Figure S6 | All | ITS2 |
| AY170855 | Supplemental Figure S6 | All | ITS2 |
| AJ400491 | Supplemental Figure S6 | All | ITS2 |
| JQ082321 | Supplemental Figure S6 | All | ITS2 |
| FR865730 | Supplemental Figure S6 | All | ITS2 |
| KF689576 | Supplemental Figure S6 | All | ITS2 |
| FR865733 | Supplemental Figure S6 | All | ITS2 |
| JN703736 | Supplemental Figure S6 | All | ITS2 |
| FR865723 | Supplemental Figure S6 | All | ITS2 |
| AJ237955 | Supplemental Figure S6 | All | ITS2 |
| JQ082331 | Supplemental Figure S6 | All | ITS2 |
| KF209348 | Supplemental Figure S6 | All | ITS2 |
| FR865735 | Supplemental Figure S6 | All | ITS2 |
| JQ082332 | Supplemental Figure S6 | All | ITS2 |
| AJ237954 | Supplemental Figure S6 | All | ITS2 |
| JQ082334 | Supplemental Figure S6 | All | ITS2 |
| JQ082323 | Supplemental Figure S6 | All | ITS2 |
| AY170857 | Supplemental Figure S6 | All | ITS2 |
| GQ375104 | Supplemental Figure S6 | All | ITS2 |
| AJ237952 | Supplemental Figure S6 | All | ITS2 |
| AY170856 | Supplemental Figure S6 | All | ITS2 |
| JX456466 | Supplemental Figure S6 | All | ITS2 |
| AY170858 | Supplemental Figure S6 | All | ITS2 |
| AJ237950 | Supplemental Figure S6 | All | ITS2 |
| JX485652 | Supplemental Figure S6 | All | ITS2 |
| JX290026 | Supplemental Figure S6 | All | ITS2 |
| JQ082326 | Supplemental Figure S6 | All | ITS2 |
| AJ237951 | Supplemental Figure S6 | All | ITS2 |
| JQ082312 | Supplemental Figure S6 | All | ITS2 |
| AJ237949 | Supplemental Figure S6 | All | ITS2 |
| AJ249511 | Supplemental Figure S6 | All | ITS2 |
| DQ417568 | Supplemental Figure S6 | All | ITS2 |
| FR865734 | Supplemental Figure S6 | All | ITS2 |
| JX046428 | Supplemental Figure S6 | All | ITS2 |
| JQ082317 | Supplemental Figure S6 | All | ITS2 |
| KF689590 | Supplemental Figure S6 | All | ITS2 |
| AJ249508 | Supplemental Figure S6 | All | ITS2 |
| KF689581 | Supplemental Figure S6 | All | ITS2 |
| KF689568 | Supplemental Figure S6 | All | ITS2 |
| FR865736 | Supplemental Figure S6 | All | ITS2 |
| JX046419 | Supplemental Figure S6 | All | ITS2 |
| AJ249510 | Supplemental Figure S6 | All | ITS2 |
| JQ315790 | Supplemental Figure S6 | All | ITS2 |
| KF689582 | Supplemental Figure S6 | All | ITS2 |
| AJ249509 | Supplemental Figure S6 | All | ITS2 |
| KF689586 | Supplemental Figure S6 | All | ITS2 |
| JQ082324 | Supplemental Figure S6 | All | ITS2 |
| JQ082318 | Supplemental Figure S6 | All | ITS2 |
| FR865725 | Supplemental Figure S6 | All | ITS2 |
| KF689578 | Supplemental Figure S6 | All | ITS2 |
| KF689585 | Supplemental Figure S6 | All | ITS2 |
| JQ315791 | Supplemental Figure S6 | All | ITS2 |
| KF689588 | Supplemental Figure S6 | All | ITS2 |
| KF689579 | Supplemental Figure S6 | All | ITS2 |
| JX014221 | Supplemental Figure S6 | All | ITS2 |
| KF689583 | Supplemental Figure S6 | All | ITS2 |
| EU729732 | Supplemental Figure S6 | All | ITS2 |
| AJ249506 | Supplemental Figure S6 | All | ITS2 |
| JQ315793 | Supplemental Figure S6 | All | ITS2 |
| KF689589 | Supplemental Figure S6 | All | ITS2 |
| JQ240279 | Supplemental Figure S6 | All | ITS2 |
| JQ082329 | Supplemental Figure S6 | All | ITS2 |
| KF209348 | Supplemental Figure S6 | All | ITS2 |
| JN660614 | Supplemental Figure S6 | All | ITS2 |
| JN660613 | Supplemental Figure S6 | All | ITS2 |
| JN660611 | Supplemental Figure S6 | All | ITS2 |
| JN660715 | Supplemental Figure S6 | All | ITS2 |
| AY461362 | Supplemental Figure S6 | All | ITS2 |
| AY819731 | Supplemental Figure S6 | All | ITS2 |
| AY461363 | Supplemental Figure S6 | All | ITS2 |
| JQ240279 | Supplemental Figure S6 | All | ITS2 |
| JN660607 | Supplemental Figure S6 | All | ITS2 |
| AM410657 | Supplemental Figure S6 | All | ITS2 |
| AM410658 | Supplemental Figure S6 | All | ITS2 |
| AM410654 | Supplemental Figure S6 | All | ITS2 |
| AM410655 | Supplemental Figure S6 | All | ITS2 |
| AM410652 | Supplemental Figure S6 | All | ITS2 |
| AM410666 | Supplemental Figure S6 | All | ITS2 |
| AM410653 | Supplemental Figure S6 | All | ITS2 |
| AM410659 | Supplemental Figure S6 | All | ITS2 |
| JN660714 | Supplemental Figure S6 | All | ITS2 |
| AM419228 | Supplemental Figure S6 | All | ITS2 |
| AJ249508 | Supplemental Figure S6 | All | ITS2 |
| AJ249510 | Supplemental Figure S6 | All | ITS2 |
| AJ249509 | Supplemental Figure S6 | All | ITS2 |
| EU878192 | Supplemental Figure S6 | All | ITS2 |
| FJ593899 | Supplemental Figure S6 | All | ITS2 |
| GQ375089 | Supplemental Figure S6 | All | ITS2 |
| GQ375093 | Supplemental Figure S6 | All | ITS2 |
| GQ375092 | Supplemental Figure S6 | All | ITS2 |
| FJ796893 | Supplemental Figure S6 | All | ITS2 |
| GQ375104 | Supplemental Figure S6 | All | ITS2 |
| GQ375096 | Supplemental Figure S6 | All | ITS2 |
| JQ615588 | Supplemental Figure S6 | All | ITS2 |
| JF835988 | Supplemental Figure S6 | All | ITS2 |
| JQ082332 | Supplemental Figure S6 | All | ITS2 |
| JQ082334 | Supplemental Figure S6 | All | ITS2 |
| JF835990 | Supplemental Figure S6 | All | ITS2 |
| JQ867365 | Supplemental Figure S6 | All | ITS2 |
| EU729730 | Supplemental Figure S6 | All | ITS2 |
| EU729729 | Supplemental Figure S6 | All | ITS2 |
| JQ867368 | Supplemental Figure S6 | All | ITS2 |
| JQ082321 | Supplemental Figure S6 | All | ITS2 |
| JQ082331 | Supplemental Figure S6 | All | ITS2 |
| JQ082323 | Supplemental Figure S6 | All | ITS2 |
| JQ082326 | Supplemental Figure S6 | All | ITS2 |
| JQ082312 | Supplemental Figure S6 | All | ITS2 |
| JQ082317 | Supplemental Figure S6 | All | ITS2 |
| JQ082324 | Supplemental Figure S6 | All | ITS2 |
| JQ082318 | Supplemental Figure S6 | All | ITS2 |
| EU729732 | Supplemental Figure S6 | All | ITS2 |
| JQ082329 | Supplemental Figure S6 | All | ITS2 |
| JN660713 | Supplemental Figure S6 | All | ITS2 |
| JN660636 | Supplemental Figure S6 | All | ITS2 |
| JN660673 | Supplemental Figure S6 | All | ITS2 |
| JN660630 | Supplemental Figure S6 | All | ITS2 |
| JN660765 | Supplemental Figure S6 | All | ITS2 |
| JN660603 | Supplemental Figure S6 | All | ITS2 |
| KF689567 | Supplemental Figure S6 | All | ITS2 |
| EU502835 | Supplemental Figure S6 | All | ITS2 |
| KF537768 | Supplemental Figure S6 | All | ITS2 |
| KF537766 | Supplemental Figure S6 | All | ITS2 |
| JX046434 | Supplemental Figure S6 | All | ITS2 |
| KF689581 | Supplemental Figure S6 | All | ITS2 |
| KF537775 | Supplemental Figure S6 | All | ITS2 |
| JX046429 | Supplemental Figure S6 | All | ITS2 |
| JN660628 | Supplemental Figure S6 | All | ITS2 |
| KF689591 | Supplemental Figure S6 | All | ITS2 |
| JN832676 | Supplemental Figure S6 | All | ITS2 |
| JN660667 | Supplemental Figure S6 | All | ITS2 |
| KF689588 | Supplemental Figure S6 | All | ITS2 |
| KF689578 | Supplemental Figure S6 | All | ITS2 |
| KF689589 | Supplemental Figure S6 | All | ITS2 |
| KF689576 | Supplemental Figure S6 | All | ITS2 |
| KF689590 | Supplemental Figure S6 | All | ITS2 |
| KF689583 | Supplemental Figure S6 | All | ITS2 |
| KF689585 | Supplemental Figure S6 | All | ITS2 |
| KF689579 | Supplemental Figure S6 | All | ITS2 |
| KF689568 | Supplemental Figure S6 | All | ITS2 |
| KF689586 | Supplemental Figure S6 | All | ITS2 |
| KF689582 | Supplemental Figure S6 | All | ITS2 |
| JX014221 | Supplemental Figure S6 | All | ITS2 |
| JF737776 | Supplemental Figure S6 | All | ITS2 |
| JX485652 | Supplemental Figure S6 | All | ITS2 |
| JX046428 | Supplemental Figure S6 | All | ITS2 |
| DQ417568 | Supplemental Figure S6 | All | ITS2 |
| DQ417571 | Supplemental Figure S6 | All | ITS2 |
| JN703736 | Supplemental Figure S6 | All | ITS2 |
| FR865735 | Supplemental Figure S6 | All | ITS2 |
| AB762692 | Supplemental Figure S6 | All | ITS2 |
| AB762691 | Supplemental Figure S6 | All | ITS2 |
| AB762693 | Supplemental Figure S6 | All | ITS2 |
| JX456465 | Supplemental Figure S6 | All | ITS2 |
| JX456466 | Supplemental Figure S6 | All | ITS2 |
| JX290025 | Supplemental Figure S6 | All | ITS2 |
| JX290026 | Supplemental Figure S6 | All | ITS2 |
| JX046419 | Supplemental Figure S6 | All | ITS2 |
| JQ043184 | Supplemental Figure S6 | All | ITS2 |
| JQ315791 | Supplemental Figure S6 | All | ITS2 |
| JQ315790 | Supplemental Figure S6 | All | ITS2 |
| JQ315793 | Supplemental Figure S6 | All | ITS2 |
| FR865661 | Supplemental Figure S6 | All | ITS2 |
| FR865723 | Supplemental Figure S6 | All | ITS2 |
| FR865687 | Supplemental Figure S6 | All | ITS2 |
| FR865730 | Supplemental Figure S6 | All | ITS2 |
| FR865736 | Supplemental Figure S6 | All | ITS2 |
| FR865734 | Supplemental Figure S6 | All | ITS2 |
| FR865725 | Supplemental Figure S6 | All | ITS2 |
| FR865601 | Supplemental Figure S6 | All | ITS2 |
| FR865733 | Supplemental Figure S6 | All | ITS2 |
| FR865674 | Supplemental Figure S6 | All | ITS2 |
